# Therapeutic targeting Tudor domains in leukemia via CRISPR-Scan Assisted Drug Discovery

**DOI:** 10.1126/sciadv.adk3127

**Published:** 2024-02-23

**Authors:** Anthony K.N. Chan, Li Han, Christopher D. Delaney, Xueer Wang, Elizaveta Mukhaleva, Mingli Li, Lu Yang, Sheela Pangeni Pokharel, Nicole Mattson, Michelle Garcia, Bintao Wang, Xiaobao Xu, Leisi Zhang, Priyanka Singh, Zeinab Elsayed, Renee Chen, Benjamin Kuang, Jinhui Wang, Yate-Ching Yuan, Bryan Chen, Lai N. Chan, Steven T. Rosen, David Horne, Markus Müschen, Jianjun Chen, Nagarajan Vaidehi, Scott A. Armstrong, Rui Su, Chun-Wei Chen

**Affiliations:** ^1^ Department of Systems Biology, Beckman Research Institute, City of Hope, Duarte, CA, USA.; ^2^ Division of Epigenetic and Transcriptional Engineering, Beckman Research Institute, City of Hope, Duarte, CA, USA.; ^3^ School of Pharmacy, China Medical University, Shenyang, Liaoning, China.; ^4^ Duke University School of Medicine, Durham, NC, USA.; ^5^ Department of Pediatrics, Dana-Farber Cancer Institute, Harvard Medical School, Boston, MA, USA.; ^6^ Department of Computational and Quantitative Medicine, Beckman Research Institute, City of Hope, Duarte, CA, USA.; ^7^ Department of Chemistry, Dartmouth College, Hanover, NH, USA.; ^8^ City of Hope Comprehensive Cancer Center, Duarte, CA, USA.; ^9^ Center of Molecular and Cellular Oncology, Yale Cancer Center, Yale School of Medicine, New Haven, CT, USA.; ^10^ Department of Cancer Biology, Lerner Research Institute, Cleveland Clinic, Cleveland, OH, USA.

## Abstract

Epigenetic dysregulation has been reported in multiple cancers including leukemias. Nonetheless, the roles of the epigenetic reader Tudor domains in leukemia progression and therapy remain unexplored. Here, we conducted a Tudor domain–focused CRISPR screen and identified SGF29, a component of SAGA/ATAC acetyltransferase complexes, as a crucial factor for H3K9 acetylation, ribosomal gene expression, and leukemogenesis. To facilitate drug development, we integrated the CRISPR tiling scan with compound docking and molecular dynamics simulation, presenting a generally applicable strategy called CRISPR-Scan Assisted Drug Discovery (CRISPR-SADD). Using this approach, we identified a lead inhibitor that selectively targets SGF29’s Tudor domain and demonstrates efficacy against leukemia. Furthermore, we propose that the structural genetics approach used in our study can be widely applied to diverse fields for de novo drug discovery.

## Teaser

CRISPR-SADD pipeline identifies SGF29 Tudor domain inhibitor for leukemia therapy.

## INTRODUCTION

Leukemia is a class of malignant blood disorders characterized by aggressive proliferation and impaired maturation of the hematopoietic stem/progenitor cells (HSPCs). Although the 5-year survival rate for leukemia has been improved from 14% in ~1960 to higher than 60% in ~2010, the overall survival of the more malignant subtypes such as acute myeloid leukemia (AML; overall survival rate < 25%) (*1*, *2*) and acute lymphoblastic leukemia [ALL; particularly the *KMT2A* (*MLL*)–rearranged subtype; overall survival rate < 20% in adult patients] (*3*) remain stunningly low. The unmet clinical needs and the lack of an effective targeted therapy emphasize the dire need for novel regimens for these malignancies. Notably, epigenetic abnormalities have been reported in multiple malignancies, including blood lineages (*4*, *5*). Thus, targeting the indispensable epigenetic circuitries represents a field of opportunity for more effective therapies in hematopoietic disorders (*6*).

Tudor domains are epigenetic reader modules that recognize chromatin modifications, particularly the methylated lysine (K) and arginine (R) (*7*, *8*). Proteins containing Tudor domains bind histone methylations at specific histone tail positions, thereby allowing interpretation of the epigenetic codes and regulation of gene expression (*9*). Similar to the well-studied bromodomains and YEATS domains (mainly recognize the acetylated or crotonylated lysines on histones) (*10*, *11*), Tudor domains are well-folded protein modules, each with a defined “aromatic cage” typically consisting of two tyrosine (Y) and one phenylalanine (F) residues for target interaction (*12*). The selective chromatin marks recognized by distinct Tudor domains represent attractive pockets for pharmaceutical targeting. However, the roles of Tudor domains in leukemia and their potential to serve as therapy targets have not been well studied.

In this study, we conducted an unbiased domain-focused CRISPR library screen (*13*) [a custom built 992–single guide RNA (sgRNA) library targeting 59 Tudor domains] and identified the requirement of the Spt-Ada-Gcn5 acetyltransferase (SAGA)–associated factor 29 (SGF29; also known as CCDC101) in leukemia initiation and maintenance. Using histone proteomics, epigenetics, and transcriptomics profiling, we revealed that SGF29, a chromatin H3K4me3 reader of the SAGA/Ada-Two-A-containing (ATAC) complexes (*14*), is essential for maintaining KAT2A/B-mediated histone H3K9 acetylation. We also utilized a high-density CRISPR tilling screen (*15*–*21*) to instruct the compound docking and develop a computer-aided drug development workflow named “CRISPR-Scan Assisted Drug Discovery” (CRISPR-SADD). This pipeline allowed us to identify the first lead inhibitor targeting SGF29’s Tudor 2 domain, which has a selective efficacy against leukemias and other types of hematopoietic malignancies.

## RESULTS

### A Tudor domain-focused CRISPR screen identifies SGF29 as a vulnerability in leukemia

**Fig. 1.**
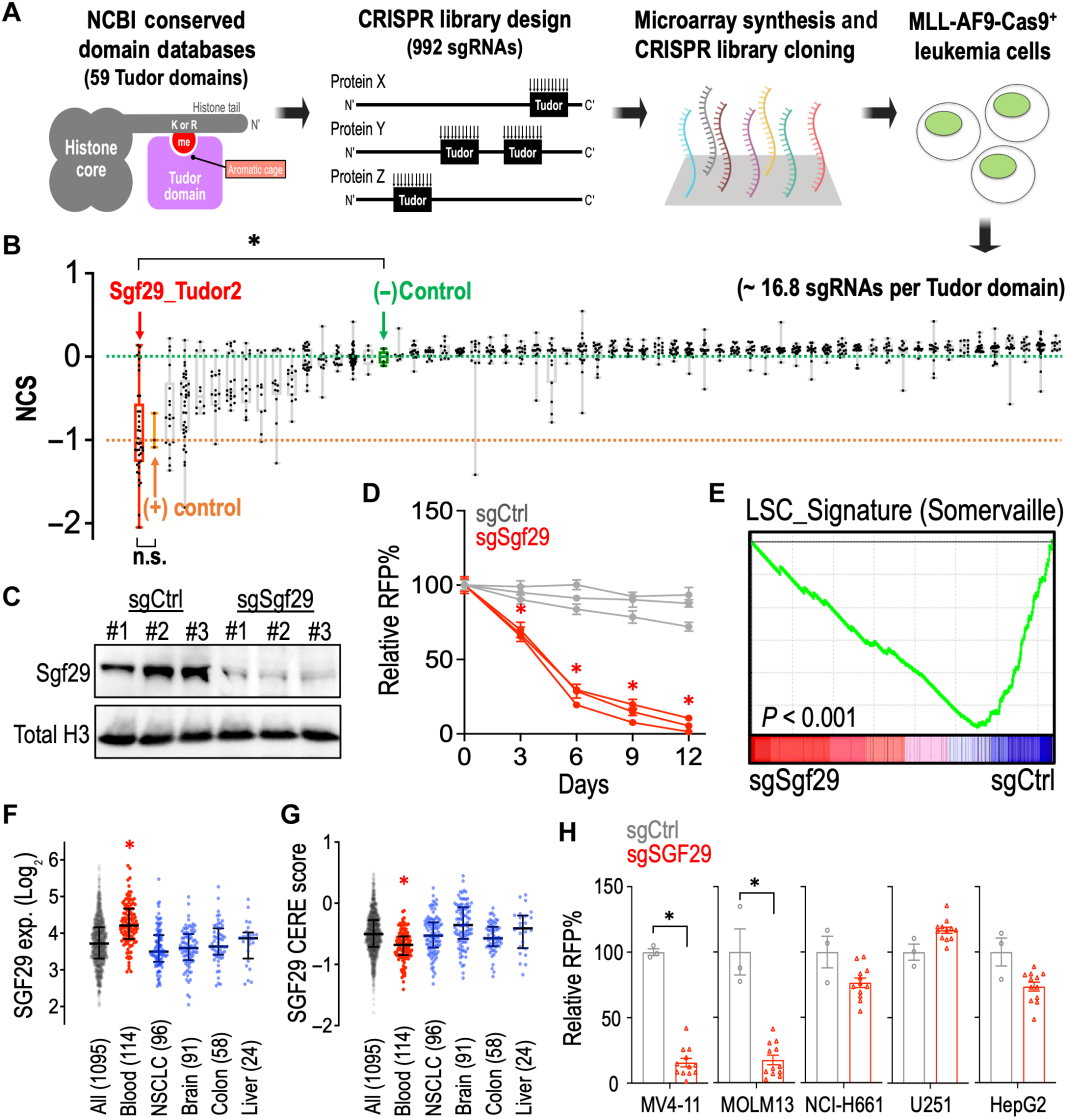
Tudor domain–focused CRISPR screen identifies the essential role of SGF29 in leukemia. (**A**) Schematic outline of a Tudor domain-focused CRISPR screen in MLL-AF9-Cas9^+^ cells. (**B**) Box whisker plots indicate the median, first and third quartiles, and the normalized CRISPR score (NCS) data range of individual sgRNA (dots) during 12 days of Tudor domain CRISPR screen culture. The median of negative controls (defined as NCS = 0.0; green dashed line) and positive controls (defined as NCS = −1.0; orange dashed line) are indicated. (**C**) Western blot of Sgf29 and histone H3 in MLL-AF9-Cas9^+^ cells transduced with sgCtrl and sgSgf29. (**D**) Growth competition assay of MLL-AF9-Cas9^+^ cells transduced with red fluorescent protein (RFP)–labeled sgCtrl (gray lines; *n* = 3 independent sgRNA sequences) and sgSgf29 (red lines; *n* = 3 independent sgRNA sequences). (**E**) RNA-seq and GSEA analyses showing changes in expression of the “LSC_Signature” (Somervaille) gene set in sgCtrl- and sgSgf29-transduced MLL-AF9-Cas9^+^ cells. Dot plots showing the (**F**) expression level and (**G**) the CRISPR gene dependency (CERE) score (right) of SGF29 in a total of human 1095 cancer cell lines tested in the DepMap consortium (Broad Institute). (**H**) Growth competition assay of Cas9-expressing MV4-11, MOLM13, NCI-H661, U251, and HepG2 cells transduced with RFP-labeled sgCtrl (*n* = 3) and sgSGF29 (*n* = 12). Data are represented as [(D) and (H)] means ± SEM and [(F) and (G)] median ± interquartile range. *P < 0.01 by two-sided Student’s *t* test. n.s., not significant.

To identify critical Tudor domains required by leukemia, we evaluated the NCBI Conserved Domains Database and summarized 59 Tudor domains in the mammalian genome (span across 36 proteins; data S1) and developed a custom CRISPR library targeting these Tudor domains with 992 sgRNAs (Fig. 1A; ~16.8 sgRNAs per Tudor domain; fig. S1 and data S2). We then delivered this library into the Cas9-expressing murine MLL-AF9 leukemia cells (a well-established AML model driven by a t(9;11) oncogenic fusion protein) (*22*) using the lentiviral transduction and compared the change of frequency of each integrated sgRNA construct in early (day 0) and late (day 12) time points. This CRISPR domain screen identified the second Tudor domain of SGF29 (SGF29_Tudor 2) (*14*), a histone H3K4me3 binding protein in the SAGA and ATAC histone modification complexes (*23*, *24*), as the top essential Tudor domain in MLL-AF9 leukemia (Fig. 1B and data S3).

To validate the library screen results, we transduced the MLL-AF9-Cas9^+^ cells with sgRNAs and found that cells transduced with sgSgf29 (Fig. 1C) were outcompeted compared to cells transduced with sgRNA targeting nonessential sequences (sgCtrl) in a flow cytometric growth competition assay (Fig. 1D and figs. S2 and S3A). Transcriptomic analysis through RNA sequencing (RNA-seq) and Gene Set Enrichment Analysis (GSEA) (*25*) revealed an attenuated leukemic stem cell (LSC) signature in the sgSgf29-targeted cells (Fig. 1E). Analysis of the cancer cell line transcriptomic (RNA-seq) and CERE score (a computational method to estimate gene-dependency levels from CRISPR-Cas9 essentiality screens) (*26*) databases (Fig. 1, F and G; total of 1095 cell lines; data source: https://depmap.org/portal/; BROAD Institute) revealed a significantly higher SGF29 expression and survival dependency in human blood malignancies (red; 114 cell lines) compared to other cancer cell types, highlighting a potential involvement of SGF29 in hematopoietic cancers. We then examined the role of SGF29 in different human cancer cell lines and observed that sgSGF29 exhibited a selective impact on the MV4-11 and MOLM13 (leukemia) over the NCI-H661 (lung carcinoma), U251 (glioblastoma), and HepG2 (hepatocellular carcinoma) cells (Fig. 1H and fig. S3A).

### SGF29 is required for in vivo leukemia development and maintenance

**Fig. 2.**
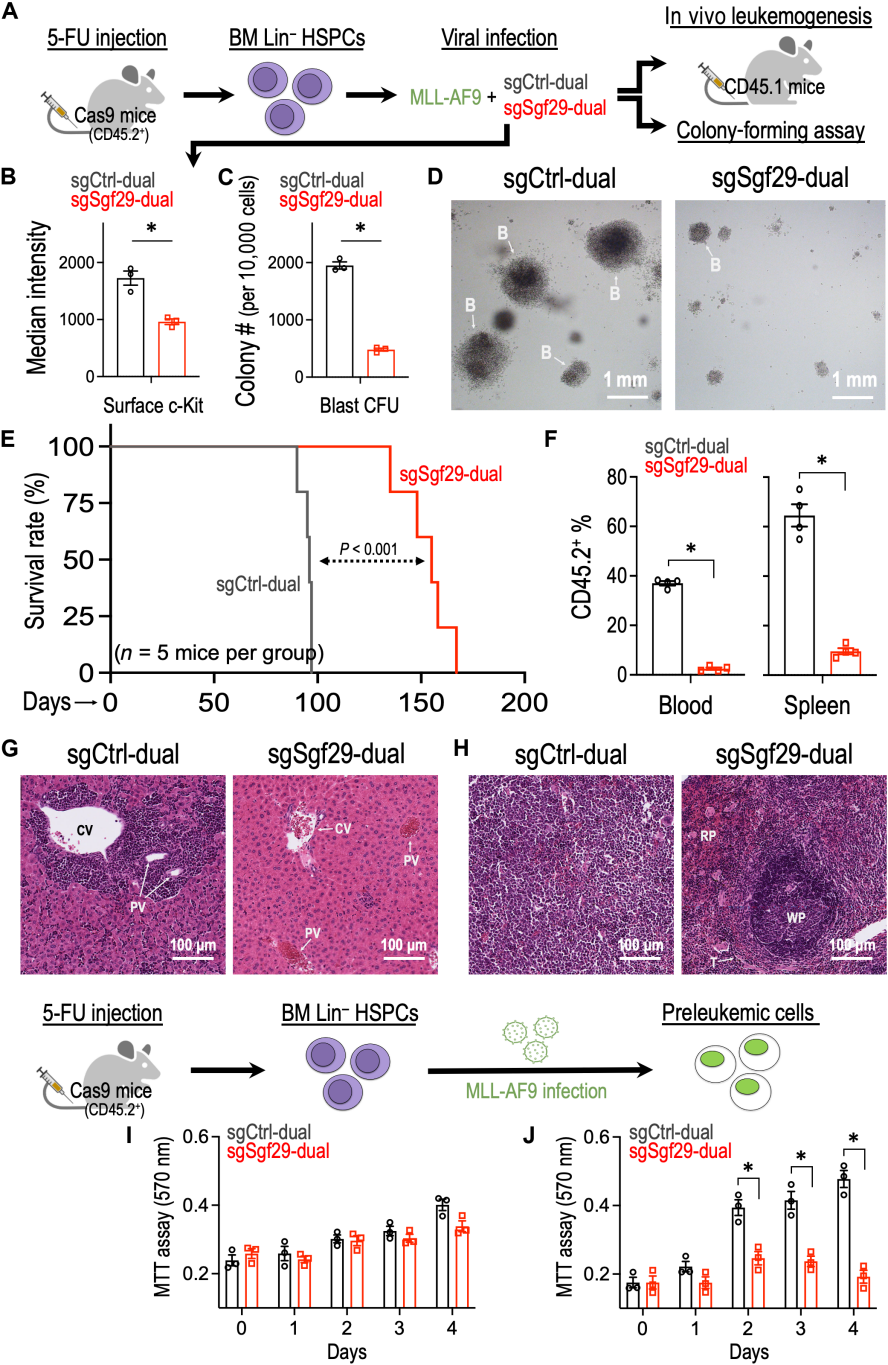
SGF29 is required for leukemia development in vivo. (**A**) Schematic outline of the primary MLL-AF9 leukemia model with Cas9-mediated Sgf29 depletion. (**B**) Flow cytometry analysis of c-Kit [phycoerythrin (PE)–Cy7] in MLL-AF9-Cas9^+^ cells transduced with sgCtrl and sgSgf29 (*n* = 3). (**C**) Effect of Sgf29 depletion on the blast-like colony forming ability of MLL-AF9–transduced preleukemic cells (*n* = 3 for each group). (**D**) Representative images of the third replating colonies from MLL-AF9–transduced preleukemic cells. B, blast-like colony. (**E**) Kaplan-Meier survival curve of recipient mice receiving MLL-AF9–transduced leukemic cells with or without Sgf29 depletion (*n* = 5 mice per group). (**F**) Percentage of CD45.2^+^ (donor) cells in the peripheral blood (left) and spleen (right) of the recipient mice (CD45.1) receiving MLL-AF9 (CD45.2^+^) transduced leukemic cells with or without Sgf29 depletion (*n* = 4 mice per group; day 90 after transplantation). The CD45.2^+^ cells represented the engraftment of leukemic MLL-AF9 cells in recipient mice. Hematoxylin and eosin stain of (**G**) liver and (**H**) spleen harvested from recipient mice receiving MLL-AF9–transduced leukemic cells with or without Sgf29 depletion. CV, central vein; PV, portal vein; WP, white pulp; RP, red pulp; T, trabecula. Effect of Sgf29 depletion on the proliferation of (**I**) BM HSPC- and (**J**) MLL-AF9–transduced preleukemic cells (*n* = 3 for each group). Data are represented as means ± SEM. *P < 0.01 by two-sided Student’s *t* test. CFU, colony-forming unit; 5-FU, 5-fluorouracil; MTT, 3-(4,5-dimethylthiazol-2-yl)-2,5-diphenyltetrazolium bromide.

To elucidate the role of SGF29 in leukemogenesis, we isolated mouse bone marrow (BM) lineage-negative (Lin^−^) HSPCs from the 5-fluorouracil (5-FU) primed Cas9-expressing mice (CD45.2^+^) (*27*) and virally transduced them with the MLL-AF9 oncogene (*22*) together with a dual sgRNA system targeting mouse Sgf29 (sgSgf29-dual) and control (sgCtrl-dual) sequences (Fig. 2A and fig. S3B) (*28*). CRISPR depletion of Sgf29 reduced the expression of c-Kit (Fig. 2B; an LSC surface marker) and diminished the capacity of MLL-AF9 to induce blast-like colonies in the replating assays (Fig. 2, C and D). We also transplanted these CD45.2^+^ preleukemic cells into the lethally irradiated CD45.1^+^ recipient mice. We showed that depletion of Sgf29 delayed the leukemia development in the recipient mice (Fig. 2E) with a decreased engraftment of CD45.2^+^ leukemic cells into peripheral blood and spleen of the recipient mice (Fig. 2F). Transduction of sgSgf29 also restrained the infiltration of leukemic blasts (cells with dark purple stained, round nuclei) into the liver/spleen and maintained the intact architecture of these organs (Fig. 2, G and H). In addition, we seeded sgCtrl-dual– or sgSgf29-dual–transduced HSPC and revealed that depletion of Sgf29 does not affect the survival of normal hematopoietic cells (Fig. 2I). On the other hand, the MLL-AF9 transduction increased the proliferation potential of the BM progenitors, and this pro-proliferative effect was impaired by CRISPR depletion of Sgf29 (Fig. 2J).

**Fig. 3.**
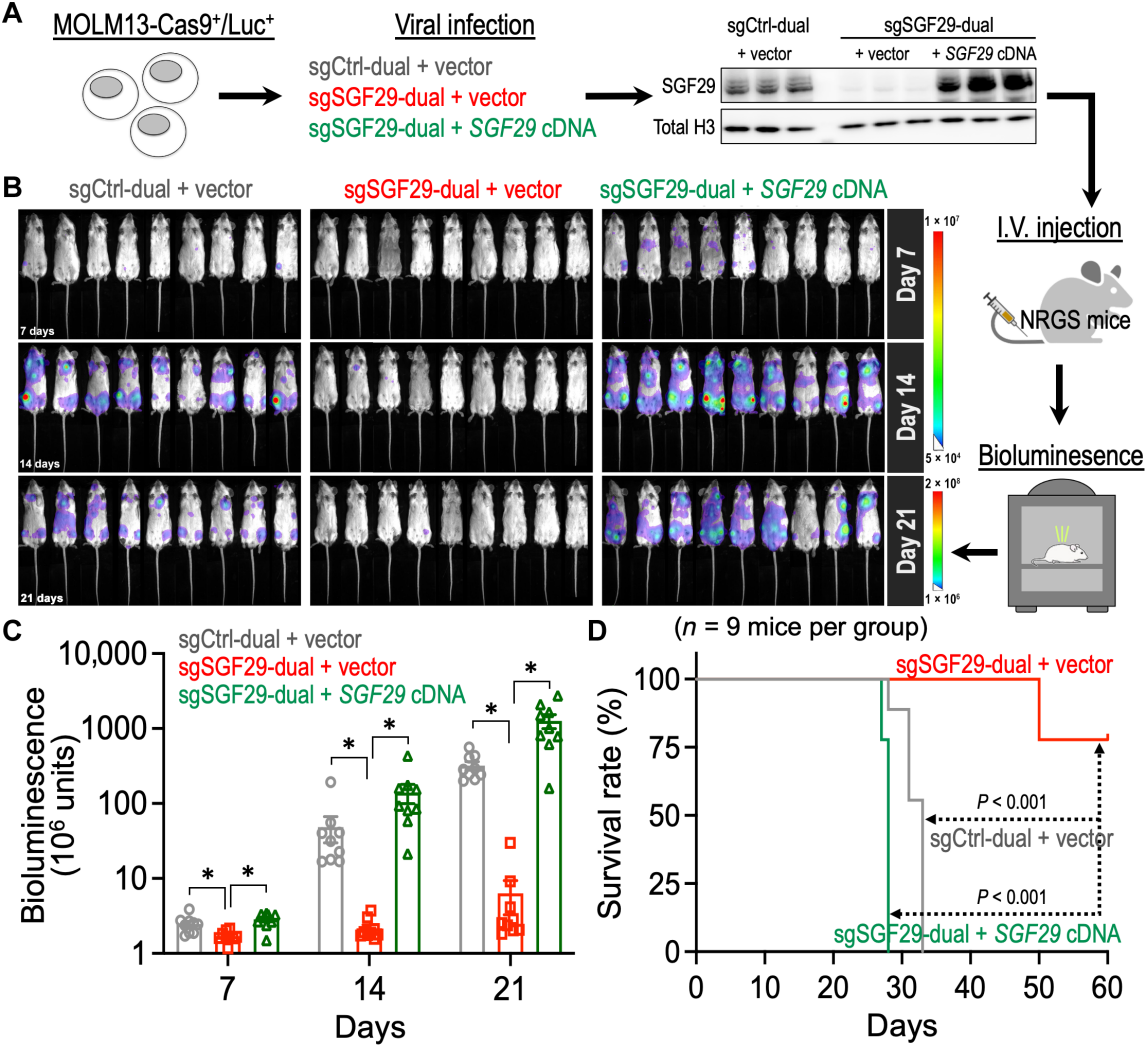
SGF29 is essential to the maintenance of human leukemia xenografts in vivo. (**A**) Schematic outline of a “human-in-mouse” leukemia xenograft model using NRGS mice (recipients) and the Cas9/luciferase-expressing human MOLM13 leukemic cells with or without SGF29 manipulation. (**B**) In vivo bioluminescent images of NRGS recipient mice transplanted with MOLM13-Cas9^+^/Luc^+^ leukemic cells with or without SGF29 manipulation (*n* = 9 mice per group). (**C**) Quantitative bioluminescent signal of NRGS recipient mice transplanted with MOLM13-Cas9^+^/Luc^+^ leukemic cells with or without SGF29 manipulation (*n* = 9 mice per group). (**D**) Kaplan-Meier survival curve of NRGS recipient mice transplanted with MOLM13-Cas9^+^/Luc^+^ leukemic cells with or without SGF29 manipulation (*n* = 9 mice per group). Data are represented as means ± SEM. *P < 0.01 by two-sided Student’s *t* test. I.V., intravenous.

To examine the impact of targeting SGF29 in the maintenance of human leukemia, we transduced a MOML13-Cas9^+^/Luc^+^ human leukemia model (*29*) with sgCtrl-dual and sgSGF29-dual (fig. S3B). We also rescued the SGF29 expression using a synthetic human *SGF29* cDNA (pLVN-hSGF29_TST) containing synonymous mutations to bypass the sgSGF29-dual targeting (Fig. 3A and fig. S4). We then transplanted these human leukemia cells into the immunodeficient NRG-SGM3 (NRGS; IMSR_JAX:024099; the Jackson Laboratory) recipient mice and monitored the leukemia progression by bioluminescence imaging (Fig. 3B). This “human-in-mouse” xenograft leukemia model revealed a notable reduction of the leukemia burden by SGF29 depletion (Fig. 3, B and C; gray versus red). In consistence with this anti-leukemia phenotype, genetic depletion of SGF29 substantially delayed leukemia onset and prolonged the overall survival of leukemia mice (Fig. 3D; gray versus red). On the other hand, ectopic expression of the synthetic *SGF29* cDNA completely reversed sgSGF29-dual–mediated anti-leukemia phenotypes (Fig. 3, B to D; green group), providing proof-of-concept evidence of targeting SGF29 in vivo to disrupt the progression/maintenance of human leukemia.

### SGF29 controls histone H3K9 acetylation and ribosomal gene expression

**Fig. 4.**
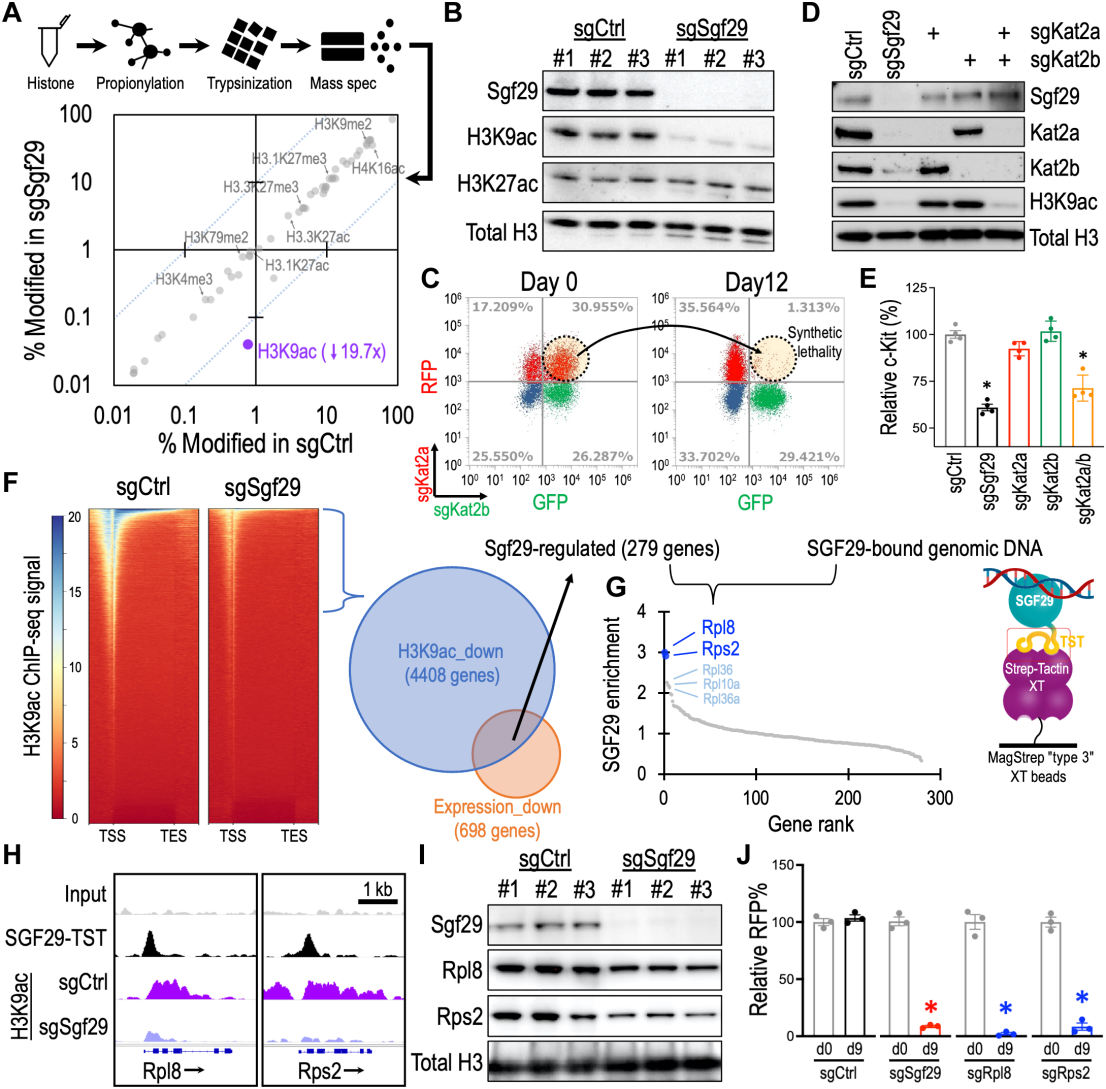
SGF29 mediates histone H3K9 acetylation and ribosomal gene expression in leukemia. (**A**) Posttranslational modification mass spectrometry of histone proteins harvested from MLL-AF9-Cas9^+^ cells transduced with sgCtrl (*x* axis) and sgSgf29 (*y* axis) for 3 days (*n* = 3). The dotted lines indicate 10-fold difference between sgSgf29 and sgCtrl. (**B**) Western blot of MLL-AF9-Cas9^+^ cells transduced with sgCtrl and sgSgf29 for 3 days. (**C**) Flow cytometry of MLL-AF9-Cas9^+^ cells transduced with sgKat2a (RFP^+^) and sgKat2b (GFP^+^) on days 0 and 12. (**D**) Western blot of MLL-AF9-Cas9^+^ cells transduced with sgCtrl, sgSgf29, sgKat2a, and sgKat2b for 3 days. (**E**) Flow cytometry of c-Kit in MLL-AF9-Cas9^+^ cells transduced with sgCtrl, sgSgf29, sgKat2a, and sgKat2b for 3 days. The relative c-Kit (%) indicates the median intensity of c-Kit (PE-Cy7) normalized to the sgCtrl group (*n* = 4). (**F**) Left: Heatmaps showing ChIP-seq signal of H3K9ac at gene coding regions from −2 kb of transcription start site (TSS) to +2 kb of transcription end site (TES) for all genes in MLL-AF9-Cas9^+^ cells transduced with sgCtrl and sgSgf29 for 3 days (*n* = 3). Right: A total of 279 Sgf29-regulated genes were identified by overlapping 4408 genes with reduced H3K9ac and 698 genes showing reduced expression in the sgSgf29-transduced MLL-AF9 leukemia. (**G**) High-throughput sequencing of genomic DNA associated with SGF29-TST identified the ribosomal genes in the Sgf29-regulated genes as SGF29-binding targets. (**H**) Distribution of SGF29-TST and H3K9ac ChIP-seq signal at the Rpl8 and Rps2 loci in MLL-AF9 leukemia. (**I**) Western blot of MLL-AF9-Cas9^+^ cells transduced with sgCtrl and sgSgf29 for 3 days. (**J**) Relative percentages of RFP^+^ cells in the MLL-AF9-Cas9^+^ cells transduced with RFP-labeled sgCtrl, sgSgf29, sgRpl8, and sgRps2 day 0 and day 9 (*n* = 3). Data are represented as means ± SEM. *P < 0.01 by two-sided Student’s *t* test.

SGF29 is a member of the SAGA/ATAC histone modification complexes (*23*, *24*). To investigate the epigenetic role of SGF29, we quantified levels of a total of 50 histone modifications (data S4) using posttranslational modification mass spectrometry (Active Motif) and observed a pronounced reduction of acetylation at histone H3 lysine 9 (H3K9ac, a histone modification associated with active gene transcription) upon Sgf29 depletion (Fig. 4A). The selective loss of H3K9ac (but not H3K27ac; another histone mark associated with transcriptional activation) in the sgSgf29 cells was also observed by immunoblotting (Fig. 4B). Because Sgf29 does not have an acetyltransferase activity, we turned our attention to two GCN5 family H3K9 acetyltransferases, Kat2a and Kat2b, that are highly associated with Sgf29 in the SAGA and ATAC complexes (fig. S5A) (*30*). We found that depletion of either Kat2a or Kat2b individually minimally affected the cellular survival (Fig. 4C; red and green populations), H3K9ac level (Fig. 4D), and the LSC marker c-Kit expression (Fig. 4E; red and green groups) in the leukemia cells. In contrast, simultaneously targeting Kat2a and Kat2b markedly eliminated the leukemic cell number (Fig. 4C; dotted circle). The sgKat2a/sgKat2b double targeting also reduced the H3K9ac level (Fig. 4D) and c-Kit expression (Fig. 4E; orange group), resembling the impact caused by sgSgf29 in these leukemia cells. We noted that depletion of Sgf29 deminished the protein level of both Kat2a and Kat2b (Fig. 4D), suggesting a role of Sgf29 in maintaining the Kat2a/2b protein stability. These results indicate a compensatory relationship between Kat2a and Kat2b and highlight the pivotal role of Sgf29 in controlling the Kat2a/2b-mediated histone H3K9 acetylation and leukemia maintenance.

On the basis of these observations, we focused on the gene loci with reduced H3K9ac [chromatin immunoprecipitation sequencing (ChIP-seq)] and expression (RNA-seq) levels in the sgSgf29 transduced MLL-AF9 leukemia and identified 279 Sgf29-regulated genes (Fig. 4F and data S5). Furthermore, we captured genomic DNA associated with SGF29-TST (Twin-Strep-Tag fusion protein) from MLL-AF9 cells using the Strep-Tactin XT beads (*31*) for high-throughput sequencing. Overlap analysis identified two ribosomal genes within the 279 Sgf29-regulated candidate genes (Rpl8 and Rps2) that are directly bound by SGF29 (Fig. 4G and data S6; cutoff by SGF29 enrichment > 2.5). Depletion of Sgf29 diminished the H3K9ac at both *Rpl8* and *Rps2* loci (Fig. 4H), which is associated with the reduced Rpl8 and Rps2 protein expression (Fig. 4I) in the MLL-AF9 cells. Last, CRISPR depletion of Rpl8 and Rps2 inhibited the MLL-AF9 cell survival, resembling the effect of sgSgf29 on these leukemia cells (Fig. 4J).

### CRISPR-SADD identifies a lead compound targeting SGF29

**Fig. 5.**
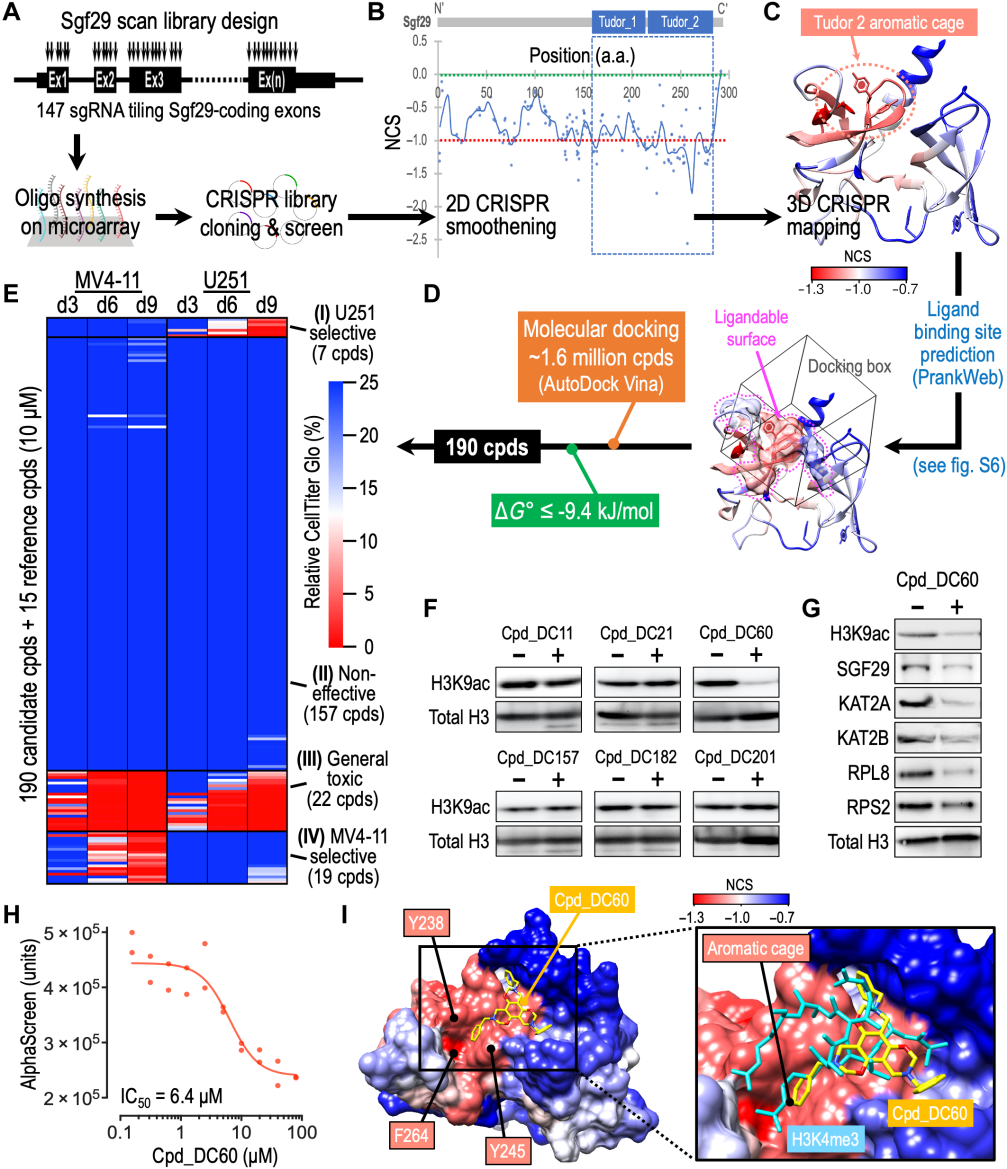
Identification of SGF29 lead inhibitor by CRISPR-SADD workflow. (**A**) Schematic outline of the Sgf29 high-density CRISPR tiling scan in MLL-AF9-Cas9^+^ cells. (**B**) Two-dimensional annotation of Sgf29 CRISPR-Scan. The blue line indicates the smoothened model of the CRISPR-Scan score derived from 147 sgRNAs (dots) targeting the coding exons of *Sgf29*. The median NCS scores of the positive control (red dotted line; defined as −1.0) and negative control (green dotted line; defined as 0.0) sgRNAs are highlighted. The blue dashed box indicates the SGF29-TTD domain. (**C**) Three-dimensional annotation Sgf29 CRISPR-Scan score relative to an x-ray crystal structural model of human SGF29-TTD (PDB ID: 3ME9). The CRISPR hypersensitive (red) aromatic cage of SGF29 Tudor_2 domain is highlighted. (**D**) Docking box (cube) defined by overlapping the PrankWeb predicted ligandable protein surface (surface contour marked by the dotted pink-line) with the CRISPR hypersensitive region of SGF29-TTD (red areas). (**E**) Heatmap showing the relative CellTiter Glo signal [% to dimethyl sulfoxide (DMSO) control] of MV4-11 and U251 cells incubated with 205 selected compounds (10 μM) for 3, 6, and 9 days. The effective killing was defined as less than 10% relative CellTiter Glo signal on day 9. Four clusters of compounds are identified, including the U251 selective (7 cpds), noneffective (157 cpds), general toxic (22 cpds), and MV4-11 selective (19 cpds) groups. (**F** and **G**) Western blot of (F) MOLM13 cells and (G) MV4-11 cells incubated with Cpd_DC60 (20 μM) for 48 hours. (**H**) Effect of Cpd_DC60 on the SGF29-TTD/H3K4me3 AlphaScreen signal. (**I**) Left: Docking simulation model of SGF29-TTD (colored by NCS) interacts with Cpd_DC60 (yellow). Right: Overlap of SGF29-TTD/H3K4me3 (cyan) cocrystal structure (PDB ID: 3ME9) with the predicted Cpd_DC60 (yellow) binding post on SGF29-TTD. The “aromatic cage” that consists of Y238, Y245, and F264 is indicated. a.a., amino acid. IC_50_, median inhibitory concentration.

To identify novel inhibitors of SGF29, we reasoned that protein surface residues that cannot tolerate the CRISPR-induced mutagenesis might indicate essential/functional positions amenable to pharmaceutical inhibition. On the basis of this, we developed a CRISPR-SADD pipeline that allows de novo identification of small molecular compounds for binding to the CRISPR hypersensitive surface areas of the targeted protein. First, we performed a high-density CRISPR gene tiling scan (*15*–*21*) in MLL-AF9-Cas9^+^ leukemia using a pool of 147 sgRNAs that targeted every “NGG” protospacer adjacent motifs (PAMs) within the endogenous *Sgf29* coding exons (Fig. 5A and data S7 and S8; targeting density of ~2.0 amino acids/sgRNA). On the bais of the local smoothen modeling (*32*) of the normalized CRISPR score (NCS), our *Sgf29* gene body scan revealed the dependency of leukemia cells to the C-terminal tandem Tudor domain (TTD) region of SGF29 (Fig. 5B, blue dashed box). We then performed a three-dimensional (3D) structural analysis of the CRISPR-Scan by mapping the smoothened NCS on a crystal structure of SGF29-TTD [Fig. 5C; Protein Data Bank (PDB) ID: 3ME9; 1.37-Å resolution] (*14*). This high-resolution structural/genetic analysis revealed the requirement of the Tudor 2 aromatic cage (dotted circle) for leukemia cell survival. We further used the PrankWeb server (*33*) to predict the surface areas suitable for binding by small molecules and overlapped with the 3D CRISPR-Scan model of SGF29-TTD to determine the druggable, CRISPR hypersensitive surface area (Fig. 5D and detailed in fig. S6; pocket B with a more depleted median NCS [−1.120] was selected for compound targeting). We then used AutoDock Vina (*34*) to dock ~1.6 million diverse compounds (selected from ZINC15 compound database; https://zinc15.docking.org) (*35*) to the CRISPR/PrankWeb-defined “docking box” and identified top 190 compounds that exhibited predicted binding free energies (Δ*G*°) ≤ −9.4 kJ/mol (data S9).

Next, we examined the response of SGF29-dependent MV4-11 leukemia cells and the SGF29-independent U251 glioblastoma cells (see Fig. 1H) to these 190 candidate compounds (plus 15 reference compounds) using a CellTiter Glo screen. This validation screen revealed four clusters of compounds (i.e., U251 selective, noneffective, general toxic, and MV4-11 selective) based on their selectivity to inhibit (i.e., relative CellTiter Glo < 10% on day 9) MV4-11 and/or U251 cells (Fig. 5E and data S9; all compounds at 10 μM). Compared to the pan-cancer inhibitors I-BET151 [inhibits the bromodomain of Bromodomain and Extra-Terminal motif (BET)] (*36*) and JQ1 (blocks BRD4 bromodomain) (*10*), we observed several known leukemia-targeting compounds (EPZ-5676, OICR-9429, A-485, and A-366) (*37*–*40*) within the 19 MV4-11 selective inhibitors (detailed in fig. S7A). On the basis of the selective killing against MV4-11 (Δ Score, i.e., differential CellTiter Glo % between MV4-11 and U251 on day 9), we then chose the top six ranked candidate compounds (Cpd_DC60, 201, 11, 157, 182, and 21) for further investigation (fig. S7B). These candidate compounds exhibit diverse structures with superior binding energy (Δ*G*° ranged from −9.5 to −10.3 kJ/mol) to the SGF29_Tudor 2 pocket compared to the endogenous ligand H3K4me3 peptide (Δ*G*° = −6.1 kJ/mol).

Because the genetic targeting of SGF29 affects the level of histone H3K9ac (Fig. 4, A and B), we used this histone modification as a biomarker to examine the candidate compounds suggested by the CRISPR-SADD pipeline. Immunoblotting revealed a notable reduction of H3K9ac level only in the Cpd_DC60 treated leukemia cells (Fig. 5F), marking Cpd_DC60 as our leading SGF29 inhibitor. Treatment of Cpd_DC60 also suppressed the expression of RPL8 and RPS2 (Fig. 5G), resembling the impact caused by SGF29 depletion in leukemia (Fig. 4I). To validate the interaction between Cpd_DC60 and SGF29, we purified the recombinant His^6^-tagged SGF29-TTD (fig. S8A; expressed using phSGF29[114 to 293 amino acids] plasmid) from *Escherichia coli* and developed an AlphaScreen assay based on the interaction of SGF29_Tudor 2 with its natural ligand H3K4me3 peptide (fig. S8B). Our results indicated that Cpd_DC60 incubation could block the AlphaScreen signal with a 50% inhibition (IC_50_) dosage of 6.4 μM (Fig. 5H). At the structural level, the “aromatic cage” of SGF29_Tudor 2 consists of three critical residues (Y238, Y245, and F264), which are essential for H3K4me3 recognition (*14*). While the Cpd_DC60 establishes a strong interaction with only one of these three aromatic residues (i.e., with Y245 through π-π stacking), our all-atom molecular dynamics simulations illustrate the favorable interactions of Cpd_DC60’s core structure with additional CRISPR hypersensitive surface areas on SGF29 (Fig. 5I, left panel) competing against the recognition site of H3K4 peptide backbone on SGF29 surface (right panel).

### Pharmaceutical targeting SGF29 inhibits leukemia progression

**Fig. 6.**
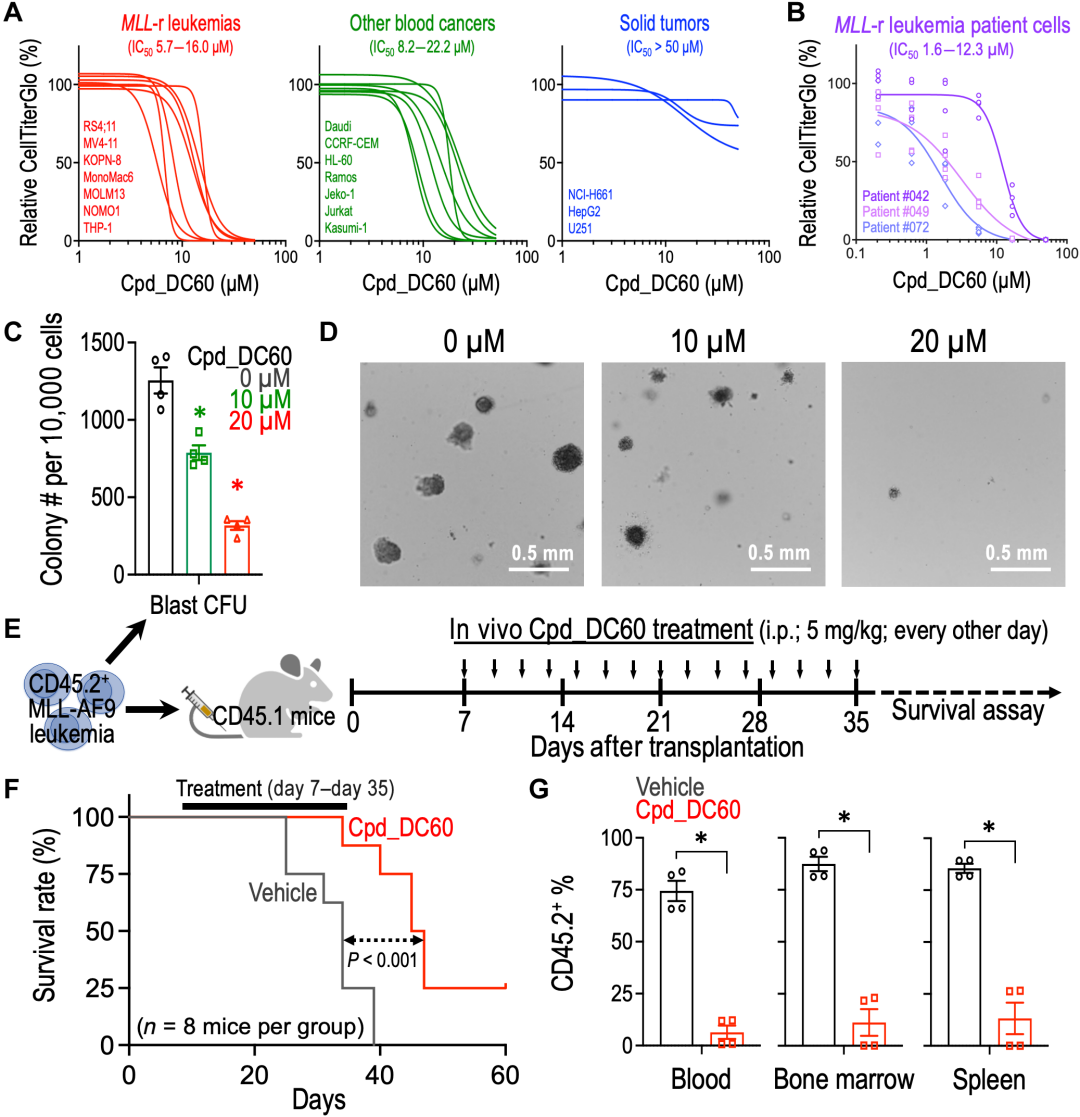
Treatment of Cpd_DC60 suppresses leukemia progression in vivo. (**A**) Effect of Cpd_DC60 on the relative CellTiter Glo signal (% to DMSO control) in *MLL*-r leukemia cells (red), non–*MLL*-r blood cancer cells (green), solid tumor cells (blue), and (**B**) *MLL*-r leukemia patient cells. Cells were incubated with Cpd_DC60 for 96 hours. The curve-fit model was performed by GraphPad Prism v9.1.1. (**C**) Effect of Cpd_DC60 on the blast-like colony-forming ability of MLL-AF9 leukemic cells (*n* = 4). (**D**) Representative images of the third replating colonies from MLL-AF9 leukemic cells treated with 0, 10, and 20 μM Cpd_DC60. (**E**) Schematic outline of the in vivo MLL-AF9 leukemia model for Cpd_DC60 treatment. (**F**) Kaplan-Meier survival curve of recipient mice receiving MLL-AF9 leukemia with or without Cpd_DC60 regimen (*n* = 8 mice per group). (**G**) Percentage of CD45.2^+^ (donor) cells in the peripheral blood, BM, and spleen of the CD45.1^+^ recipient mice with or without Cpd_DC60 treatment (*n* = 4 mice per group; day 36 after transplantation). The CD45.2^+^ cells represented the engraftment of leukemic MLL-AF9 cells in recipient mice. Data are represented as means ± SEM. *P < 0.01 by two-sided Student’s *t* test. i.p., intraperitoneal.

To examine the selectivity of SGF29 inhibition against different blood cancer types, we performed Cpd_DC60 titration experiments in *MLL*-r leukemia (Fig. 6A, red; seven cell lines), non–*MLL*-r blood cancer (green; seven cell lines), and solid tumor (blue; three cell lines) cells. We observed that the tested solid tumor cells were substantially less sensitive to the Cpd_DC60 treatment (blue; IC_50_ > 50 μM) as compared to the *MLL*-r leukemia cells (red; IC_50_: 5.7 to 16.0 μM). The non–*MLL*-r blood cancer cells (including AML, ALL, and lymphoma) exhibited comparable IC_50_ values (green; 8.2 to 22.2 μM) to the *MLL*-r leukemia cells, expanding the utility of Cpd_DC60 as a therapeutic agent for a broader spectrum of hematopoietic cancers. Furthermore, to enhance the clinical significance of the study, we tested the sensitivity of three *MLL*-r leukemia patient cell samples to Cpd_DC60. These patient samples were previously reported with well-defined mutational profiles characterized by FoundationOne Heme test (table S1) (*41*). Our result revealed a range of IC_50_ from 1.6 to 12.3 μM Cpd_DC60 on these patient leukemia cells (Fig. 6B), which is comparable to the IC_50_ observed from the human leukemia cell line models shown in Fig. 6A.

To examine the effect of Cpd_DC60 on the LSCs, we performed colony forming assay and found that Cpd_DC60 diminished the capacity of the secondarily transplanted MLL-AF9 leukemic cells to produce blast-like colonies (Fig. 6, C and D). We then transplanted these secondary MLL-AF9 leukemia (CD45.2^+^) into the sublethally irradiated CD45.1^+^ recipient mice to elucidate the efficacy of Cpd_DC60 in vivo (Fig. 6E). We showed that Cpd_DC60 regimen delayed the leukemia development in the recipient mice (Fig. 6F) with a decreased engraftment of CD45.2^+^ leukemic cells into their peripheral blood, BM, and spleen (Fig. 6G and fig. S9). We did not observe an obvious organ toxicity, hematopoietic defect, or reduced body weight in the Cpd_DC60-treated mice (fig. S10), providing proof-of-concept evidence of therapeutic targeting SGF29 in vivo for leukemia treatment.

### CRISPR-SADD pinpoints critical protein surface pockets amendable to therapeutic targeting

**Fig. 7.**
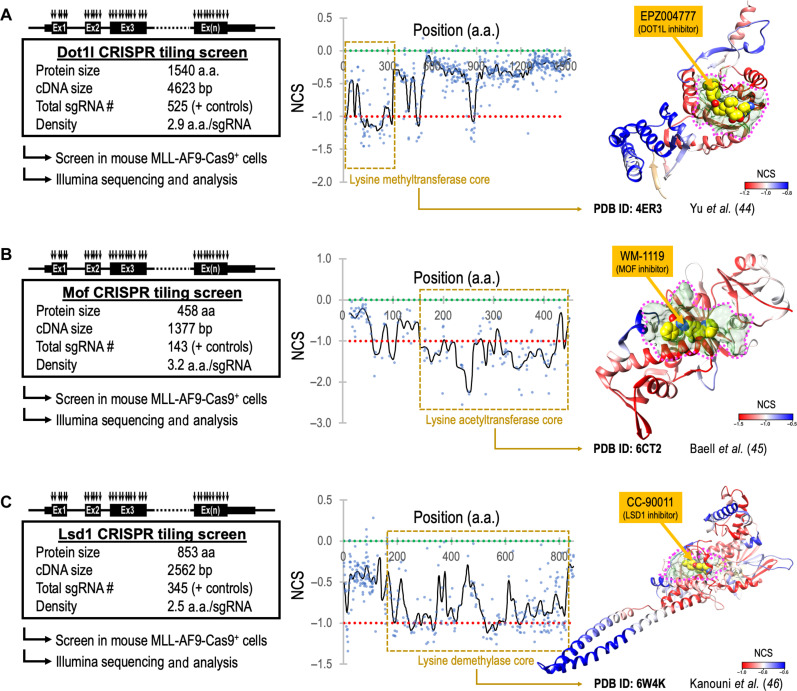
CRISPR-SADD evaluation of therapeutic target genes in leukemia. High-density CRISPR gene tiling scan of (**A**) Dot1l, (**B**) Mof, and (**C**) Lsd1 in MLL-AF9-Cas9^+^ leukemia. Left: Schematic outline of the CRISPR library designs and screens in MLL-AF9-Cas9^+^ cells. Middle: Two-dimensional annotation of CRISPR tiling scans. The black lines indicate the smoothened model of the CRISPR-Scan score derived from individual sgRNAs (dots). The median NCS scores of the positive control (red dotted line; defined as −1.0) and negative control (green dotted line; defined as 0.0) sgRNAs are highlighted. The brown dashed box indicates the catalytic core domains. Right: Three-dimensional annotation of CRISPR-Scan score relative to the x-ray crystal structural model of human DOT1L (PDB ID: 4ER3), MOF (PDB ID: 6CT2), and LSD1 (PDB ID: 6W4K). The CRISPR hypersensitive surface pockets amendable to small molecular binding (pink dotted areas) were highlighted. EPZ004777, WM-1119, and CC-90011 are cocrystallized inhibitors of DOT1L, MOF, and LSD1, respectively.

To examine the utility of CRISPR-SADD as a generally applicable tool for pharmaceutical development, we selected additional leukemia therapeutic target proteins (DOT1L, MOF, and LSD1) (*5*, *42*, *43*) that their drug-protein binding has been previously defined by cocrystallization (*44*–*46*). Specifically, we cloned three CRISPR libraries (Fig. 7, left panels), each scanned the coding exons of mouse *Dot1l* (also known as *Kmt2a*; 525 sgRNAs), *Mof* (also known as *Kat8*; 143 sgRNAs), and *Lsd1* (also known as *Kdm1a*; 345 sgRNAs). We then performed the high-density CRISPR gene tiling scans in MLL-AF9-Cas9^+^ leukemia (data S10; target density ranged from 2.5 to 3.2 amino acids per sgRNA) and aligned the smoothened NCS to the 2D peptide positions of each targeted protein (Fig. 7, middle panels). Furthermore, we used the CRISPR-SADD pipeline to highlight the CRISPR hypersensitive surface pockets amendable to small molecular binding (Fig. 7, right panels; pink dotted areas). We found the cocrystallized inhibitors (EPZ004777 for DOT1L, WM-1119 for MOF, and CC-90011 for LSD1) (*44*–*46*) all localized within the CRISPR-SADD pocket of their target proteins, validating the potential of CRISPR-SADD in defining the critical protein surface pockets for pharmaceutical development.

## DISCUSSION

Aberrant accumulation of H3K9ac has been reported at leukemic gene loci to drive uncontrollable expression of the oncogenic program (*5*). Recent studies further revealed the role of H3K9ac in recruiting the YEATS domain-containing superelongation factors ENL/AF9 for oncogenic gene expression in leukemias (*47*, *48*). Targeting the GCN5 family H3K9 acetyltransferases (including KAT2A and KAT2B) in the mammalian genome, thus represents a promising strategy for leukemia therapy (*49*). Nonetheless, our study revealed a compensatory relationship between KAT2A and KAT2B in maintaining H3K9ac, allowing leukemia cells to escape the single KAT2 gene targeting (Fig. 4, C and D). To this end, prior studies observed an evolutionarily conserved role of SGF29 in connecting the H3K4 methylations with KAT2A/2B-mediated H3K9 acetylation (*14*, *23*, *50*, *51*). Evaluation of published ChIP-seq datasets (*5*, *52*) revealed a high concordance between the distribution of H3K9ac and H3K4me3 [fig. S11; both annotate the actively transcribed gene transcription start site (TSS) regions], indicating a collaborative function between these histone posttranslational modifications.

Our histone mass spectrometry revealed a precise elimination of H3K9ac by targeting a single gene SGF29 (Fig. 4A), indicating the utility of SGF29 to serve as a selective and perhaps more effective target for H3K9ac blockade and leukemia therapy. Our RNA-seq data indicated that depletion of SGF29 did not abolish the mRNA of KAT2A and KAT2B. We also did not observe the binding of SGF29 at the *KAT2A* and *KAT2B* loci (ChIP-seq), suggesting that SGF29 regulates KAT2A/B protein level via a transcriptional independent manner. Cpd_DC60 treatment (blocks SGF29 chromatin binding domain) also reduced the SGF29 protein level in leukemic cells (Fig. 5G). Similarly, the level of KAT2A and KAT2B protein was also reduced. On the basis of these observations, we speculate that SGF29-mediated recruitment of KAT2A/B to chromatin might be required for stabilization of SGF29-KAT2A/B complex. Notably, depletion of SGF29 resulted in reduced H3K9ac and ribosomal proteins (RPL8 and RPS2) in SGF29-dependent (MOLM13 leukemia) but not in SGF29-independent (U251 glioblastoma) cells (fig. S12A). We noted that depletion of SGF29 impairs the protein level of both H3K9 acetyltransferases (KAT2A and KAT2B) in MOLM13 cells. In contrast, the impact of sgSGF29 on KAT2B protein level was less pronounced in U251 cells, and the level of H3K9ac and RPL8/RPS2 proteins was maintained. In line with this observation, we observed a selective loss of H3K9ac at *RPL8* and *RPS2* loci only in the SGF29-dependent MOLM13 cells upon sgSGF29 transduction (fig. S12B). These results highlighted a distinct regulation of H3K9ac in diverse cancer cell types, perhaps with an SGF29-independent usage of KAT2B in the sgSGF29-resistant cells. Notably, we found that the dependency of cancer cells on *SGF29* is highly correlated with the SAGA-specific members (fig. S5B, dark yellow; clustered between #3 and #220 ranked genes with the Pearson coefficient scores between 0.681 and 0.150; except *ENY2*). On the other hand, the codependency between *SGF29* and the ATAC-specific members (dark blue; ranged from #350 to #15497 ranked genes) exhibited weaker Pearson coefficient scores (Pearson coefficient < 0.127), suggesting a dominant role of SGF29 through its participation in the SAGA complex.

We also demonstrate that SGF29 contributes to the expression of ribosomal genes (RPL8, RPS2, etc.), expanding the impact of the H3K9ac epigenetic network on protein translational regulation. Notably, ectopic expression of RPL8 and RPS2 was insufficient to reverse the impact triggered by sgSGF29, suggesting additional factors downstream of SGF29 are likely required for leukemia maintenance. We observed a significant enrichment (*P* < 10^−17^) of ribosomal genes (data S6; 19 ribosomal genes labeled in green) within the SGF29-regulated gene list. Similar to the ChIP-seq profiles at the *Rpl8* (rank #1) and *Rps2* (rank #2) loci (shown in Fig. 4H), the H3K9ac level and gene expression of *Rpl36* (rank #3), *Rpl10a* (rank #4), and *Rpl36a* (rank #5) were also highly dependent on SGF29 (fig. S13A). These observations highlighted the potential of SGF29 to serve as a master regulator of ribosomal gene expression. Notably, the connection between SGF29 and leukemia progression (also SGF29 to ribosome) was not reported before. Furthermore, we examined the SGF29 and H3K9ac ChIP-seq profiles at the *Hoxa* locus, which contains a cluster of homeotic genes that are highly associated with leukemogenesis. We observed a pronounced H3K9ac deposition at the core region of the *Hoxa* cluster genes (fig. S13B, green dotted box). CRISPR depletion of SGF29 reduced the H3K9ac at this locus, which is associated with reduced gene expression in this region (e.g., *Hoxa9* and *Hoxa11*). However, we did not observe an enriched SGF29 binding signal at this locus, suggesting that the effect of SGF29 on *Hoxa* gene regulation might go through an indirect mechanism. We speculate that the impact of sgSGF29 on the global H3K9ac level could profoundly affect the epigenome beyond the direct chromatin binding targets of SGF29.

Traditional drug development benefits from known functional domains for therapeutic inhibition; however, it generally restricts the existing inhibitors to target the well-characterized enzymatic pockets, which often limited the selectivity due to the high homology shared across the gene family. Our study, on the other hand, highlighted that CRISPR gene tiling could provide a platform for pharmaceutical development, circumventing the need for prior knowledge of protein functional regions. Combined with the in silico ligand binding site prediction (e.g., PrankWeb server), compound docking (e.g., AutoDock Vina), and molecular dynamics simulations (e.g., GROMACS), our CRISPR-SADD workflow enables de novo identification of lead compounds that block the CRISPR hypersensitive pockets of the difficult-to-drug proteins (including the nonenzymatic chromatin readers, transcription factors, complex adaptors, etc.). We foresee the combination of CRISPR-SADD with the artificial intelligence–driven structural prediction (e.g., AlphaFold database) (*53*) will speed up the therapeutic development of previously untreatable diseases. We also envision CRISPR-SADD to benefit the development of proteolysis-targeting chimera based (*54*) and other types of the modern therapeutics by targeting the drugs to the CRISPR hypersensitive surface regions (not tolerated to genetic mutations), as this strategy could avoid the evolution of drug-resistant mutations that prohibit drug/target interaction.

In summary, our study highlighted that SGF29-mediated H3K9 acetylation could serve as a leukemia-selective therapeutic target. Disruption of SGF29 (via genetic suppression or the Tudor domain inhibition) suppresses leukemia progression in vitro and in vivo, providing critical rationales toward an effective therapy against hematopoietic cancers, including the more malignant subtypes such as the leukemias with *MLL* gene translocations. We foresee the chemical derivatives of Cpd_DC60 to improve the potency and in vivo bioavailability of this lead compound. We also envision that the “serial CRISPR screen” and the CRISPR-SADD approach demonstrated in our study will be highly applicable to other studies in diverse fields.

## MATERIALS AND METHODS

### Cell culture

Mouse MLL-AF9 leukemic cells were generated by transformation of mouse BM Lin^−^ cells with retrovirus expressing MLL-AF9 fusion protein and transplanted into sublethally irradiated recipient mice as described previously (*5*). The leukemic blasts harvested from the diseased mice were cultured in vitro in Iscove's modified Dulbecco's medium (IMDM) (Gibco) plus 15% fetal bovine serum (FBS) supplemented with murine SCF (20 ng/ml; PeproTech), murine interleukin-3 (IL-3) (10 ng/ml; PeproTech), and murine IL-6 (10 ng/ml; PeproTech). Human cell lines MV4-11, MOLM13, KOPN-8, RS4;11, MonoMac6, NOMO1, THP-1, Daudi, CCRF-CEM, HL-60, Ramos, Jeko-1, Jurkat, and Kasumi-1 cells were maintained in RPMI (Gibco) supplemented with 10% FBS. Human cell lines NCI-H661, U251, and HepG2 were cultured in Dulbecco’s modified Eagle’s medium (DMEM)(Gibco) supplemented with 10% FBS. All cell culture medium contained l-glutamine (2 mM; Gibco), penicillin (100 U/ml; Gibco), streptomycin (100 μg/ml; Gibco), and plasmocin (0.5 μg/ml; InvivoGen). All cells were cultured in a 37°C incubator with 5% CO_2_. Cells stably expressing the Cas9 endonuclease were established via transduction of lentiCas9-Blast (52962, Addgene) lentivirus and selected by blasticidin (Gibco).

### CRISPR library, single, and dual sgRNA construction

CRISPR sgRNAs were selected using the Genetic Perturbation Platform (Broad Institute) (*55*). For the Tudor domain–focused CRISPR library, 992 sgRNA sequences targeting the 59 Tudor domains (across 36 Tudor domain–containing mouse genes) were designed (data S1 and S2). For the gene tiling scan CRISPR libraries, sgRNA sequences targeting every PAM within the target gene (*Sgf29*, *Dot1l*, *Mof*, and *Lsd1*) coding exons were designed. Individual sgRNA selected for validation experiments are listed in fig. S3. Briefly, guide RNA oligos were synthesized by microarray (CustomArray; for library cloning) or individual oligosynthesis (Integrated DNA Technologies; for single sgRNA) and cloned into the ipUSEPR lentiviral sgRNA vector [hU6-driven sgRNA coexpressed with EF-1a–driven red fluorescent protein (RFP) and puromycin-resistance gene] using the Bsm BI (New England Biolabs) restriction sites (fig. S1A) (*15*). String assembly gRNA cloning (STAgR) (*28*) was used as a cloning method to clone two sgRNAs into Bsm BI–digested ipUSEPR using NEBuilder HiFi DNA Assembly Kit (E5520, New England Biolabs). Briefly, the STAGR_gRNAScaffold_mU6 plasmid (102844, Addgene) was used as the DNA template source to polymerase chain reaction (PCR)–amplify guide RNA scaffold and mouse U6 promoter cassette using Q5 Hot Start High-Fidelity 2X Master Mix (M0494S, New England Biolabs). The final plasmids were validated via Sanger sequencing (Eton Bioscience). All molecular cloning was performed using the NEB 5-alpha Competent *E. coli* (C2987H, New England Biolabs).

### Lentiviral production and transduction

Lentiviruses were produced in human embryonic kidney–293 cells (CRL-1573, American Type Culture Collection) with the help of the packaging plasmids pPAX2 (12260, Addgene) and pMD2.G (12259, Addgene). The lentiviral plasmid, pPAX2, and pMD2.G were mixed in a 1:1:1 ratio in the Opti-MEM medium (31-985-062, Gibco) in the presence of polyethyleneimine (50 μg/ml; PRIME-P100-100MG, Serochem LLC). After transfecting HEK293 for 24 hours, the medium supernatant was aspired and replaced with the fresh DMEM medium. Then, the transfected cells were allowed to grow for 48 hours to produce lentiviruses. Subsequently, the viral supernatants were incubated with 10% polyethylene glycol (BP233-1, Thermo Fisher Scientific) at 4°C overnight and centrifuged (3000*g*, 4°C, 30 min) to collect the precipitated viral particles. After that, the viral pellets were resuspended with an appropriate medium (e.g., DMEM, IMDM, or RPMI), aliquoted, and kept at −80°C. For lentiviral transduction of suspension cells, the cells were centrifuged at 1000*g* at 37°C for 1 hour in the presence of polybrene (10 μg/ml; TR1003G, MilliporeSigma).

### Tudor domain–focused CRISPR library screen

The Tudor domain–focused CRISPR library (992 sgRNAs targeting 56 Tudor domains) was delivered into the MLL-AF9-Cas9^+^ cells (*15*). Briefly, cells were transduced with the CRISPR library lentiviruses at ~15% infection (monitored by flow cytometry for RFP expression; three replicates) and selected by puromycin (2 μg/ml; Gibco). The library-transduced cells were subcultured every 3 days for a total of 12 days. At the start (day 0) and end (day 12) time points, 1 million cells from each screen culture were collected. The integrated sgRNA in each sample was PCR-amplified (NEBNext Ultra II Q5, New England Biolabs) using primers DCF01 5′-CTTGTGGAAAGGACGAAACACCG-3′ and DCR03 5′-CCTAGGAACAGCGGTTTAAAAAAGC-3′ for high-throughput sequencing (NextSeq550, Illumina). To quantify sgRNA reads, 20-nucleotide sequences that matched the sgRNA backbone structure (5′-CACCG and GTTT-3′) were extracted and mapped to the library sgRNA sequences using Bowtie2. The frequency for individual sgRNAs was calculated as the read counts of each sgRNA divided by the total read counts matched to the library. The NCS was defined as a log_10_ fold change in the frequency of individual sgRNAs between the start (day 0) and end (day 12) of the screened samples and normalized by the median score of the negative control sgRNA (defined as 0.0; sgRNA targeting nonessential sequences) and the median score of the positive control sgRNA (defined as −1.0; sgRNA targeting *Rpa3*) within the screen data. The candidate Tudor domains were ranked by the median NCS score of each domain (~16.8 sgRNAs per domain).

### Cloning, expression, and purification of human SGF29 and its TTD

To clone the lentiviral pLVN-hSGF29_TST for expression in mammalian cells, the full-length coding sequence of human *SGF29* was first in silico codon–optimized using CLC Genomics Workbench (QIAGEN) to introduce synonymous mutations, therefore, could bypass the sgSGF29 targeting (fig. S4A). We then chemically synthesized the optimized *SGF29* cDNA using gBlock Gene Fragments (Integrated DNA Technologies) and cloned it into the lentiviral pLVN vector (LentiV_Neo; 108101, Addgene; EF-1a–driven transgene coexpressed with Neomycin-resistance gene) (*56*) together with a C-terminal TST using the NEBuilder HiFi DNA Assembly Cloning Kit (New England Biolabs). The final plasmids were validated via Sanger sequencing (Eton Bioscience). All molecular cloning was performed using the NEB 5-alpha Competent *E. coli* (C2987H; New England Biolabs).

To clone the phSGF29[114 to 293 amino acids] for expressing the recombinant peptide in *E. coli*, the optimized *hSGF29* cDNA fragment was PCR-amplified (primers GA_3ME9_f: 5′-AGAACCTGTACTTCCAATCCATGCGTCGTGGTGTTCT-3′ and GA_uniR: 5′-CGGAGCTCGAATTCGGAT-3′) and cloned into the pNIC28-Bsa4 vector (26103, Addgene), resulting an SGF29’s TTD (SGF29-TTD; residues Met^114^ to Lys^293^; UniProt ID: Q96ES7; 22.9 kDa) sequence with an N-terminal hexahistidine tag (His^6^-tag). For recombinantly expressing SGF29-TTD, the phSGF29[114 to 293 amino acids] plasmid was first transformed into *E. coli* (BL21-CodonPlus-RIL; 230240, Agilent Technologies) in the presence of ampicillin (100 μg/ml) and chloramphenicol (50 μg/ml). The transformed *E. coli* was scaled up to 3-liter liquid cultures in Terrific Broth (BP9728-500, Thermo Fisher Scientific) at 37°C until the optical density at 660 nm reached 0.8. The expression of recombinant SGF29-TTD was induced by adding 0.1 mM isopropyl-β-d-thiogalactopyranoside (R0392, Thermo Fisher Scientific) at 16°C for 24 hours. The *E. coli* pellet was collected by centrifugation (8000*g*, 4°C, 5 min) and sonicated (50% amplitude; 5-s bursts interrupted by 5-s pauses for 60 cycles) on ice in the presence of 500 U of Benzonase (70664, MilliporeSigma) and cOmplete Protease Inhibitor Cocktail (04693159001, Roche). The cell lysate was clarified by centrifugation (10,000*g*, 4°C, 10 min) and filtering through 0.45-μm syringe filters (SLHV033NS, MilliporeSigma). The clarified cell lysate was first purified by immobilized metal affinity chromatography (IMAC) with a HisTrap HP column (5 ml, 95056-204, GE Healthcare-Cytiva) and then by size exclusion chromatography (SEC) with a HiLoad 16/600 Superdex 75-pg column (71002-666, GE Healthcare-Cytiva) using an ÄKTA start protein purification system (GE Healthcare-Cytiva). The eluted protein was stored in SEC buffer [50 mM Hepes, 100 mM NaCl, and 5 mM tris(2-carboxyethyl)phosphine (TCEP), pH 7.4]. The purified SGF29-TTD was checked by SDS–polyacrylamide gel electrophoresis and SimplyBlue (LC6060, Thermo Fisher Scientific) staining. At the final step, purified SGF29-TTD was concentrated by VIVASPIN 20 (10 kDa molecular weight cutoff, polyethersulfone; 28-9323-60, GE Healthcare-Cytiva). The concentrated SGF29-TTD protein was aliquoted, frozen by liquid nitrogen, and kept at −80°C.

### Western blotting

Cells were harvested and lysed in lithium dodecyl sulfate (LDS) sample buffer (Invitrogen) at 5 million cells/ml, separated electrophoretically using Bolt 4 to 12% bis-tris plus gels (Invitrogen), and transferred onto polyvinylidene difluoride (PVDF) membranes (0.2 μm in pore size) using PVDF Mini Stacks and iBlot 2 (Invitrogen). Membranes were immersed in 5% bovine serum albumin (Thermo Fisher Scientific) and then probed with primary antibodies against SGF29 (1:1000; HPA052590, Sigma), H3K9ac (1:1000; 61663, Active Motif), H3K27ac (1:1000; 91193, Active Motif), KAT2A (1:1000; 3305S, Cell Signaling Technology Inc.), KAT2B (1:1000; 3378S, Cell Signaling Technology Inc.), RPL8 (1:1000; HPA050165, Sigma), RPS2 (1:1000; PIPA530160, Thermo Fisher Scientific), and Histone H3 (1:1000; 4499, Cell Signaling Technology) at 4°C overnight. After washing, the membranes were incubated with horseradish peroxidase–conjugated goat anti-rabbit (1:200,000; 31460, Invitrogen), anti-mouse (1:200,000; 31430, Invitrogen), and anti-rat (1:200,000; 31470, Invitrogen) immunoglobulin G antibodies at room temperature for 1 hour. Chemiluminescent signals were developed using the SuperSignal West Femto Substrate (Thermo Fisher Scientific) and detected using a ChemiDoc imaging system (Bio-Rad).

### Flow cytometric assays

For competition cell culture assays, Cas9-expressing cells were transduced with the ipUSEPR (RFP^+^) or pLKO5.sgRNA.EFS.GFP (GFP^+^; 57822, Addgene) sgRNA constructs in 96-well plates at ~50% infection. Relative RFP% refers to percentages of RFP^+^ cells over time after lentiviral infection, which was normalized to the RFP^+^% on day 0 (i.e., 48 hours after the lentiviral infection). For cell surface marker detection, mouse MLL-AF9-Cas9^+^ cells transduced with ipUSEPR sgRNA constructs (RFP^+^) were stained by rat anti-mouse c-kit [phycoerythrin (PE)–Cy7; 1:200; 25117181, Invitrogen], CD45.1 (allophycocyanin; 1:200; 17-0453-82, eBioscience), and CD45.2 [fluorescein isothiocyanate (FITC); 1:200; 17-0454-82, eBioscience] antibodies at 4°C for 30 min. Live cells were defined by 4′,6-diamidino-2-phenylindole (D1306, Invitrogen) dye exclusion. Data were obtained by high-throughput flow cytometry using an Attune NxT flow cytometer with an autosampler (Thermo Fisher Scientific).

### Transcriptomic analysis

For RNA-seq, total RNA was extracted using the RNeasy Mini Kit (74104, QIAGEN) and submitted for mRNA library prep and sequenced by a NovaSeq 6000 [paired-end 150 base pair (bp); ~20 million reads per sample] at Novogene Inc. Raw sequence reads were mapped to the mouse genome (mm10) using STAR v2.6.1d and quantified using featureCounts v1.6.4. The raw counts were then normalized using the trimmed mean of *M* values method and compared using the Bioconductor package “edgeR.” GSEA was performed using the GSEA v4.1.0 (Broad Institute).

### Mouse MLL-AF9 leukemia, colony-forming assay, and in vivo leukemogenesis

For the mouse MLL-AF9 in vivo leukemogenesis model, 6- to 8-week-old Cas9-expressing donor mice [strain ID: Gt(ROSA)26Sor^tm1.1(CAG-cas9*,-EGFP)Fezh^/J; RRID: IMSR_JAX:024858, the Jackson Laboratory] (*27*) and B6.SJL CD45.1^+^ recipient mice (B6.SJL-*Ptprc^a^Pepc^b^*/BoyCrCrl; Charles River Laboratories) were used. To isolate the Lin^−^ HSPCs from the BM, the 4- to 6-week-old Cas9-expressing donor mice (CD45.2^+^) were administrated with 5-FU (150 mg/kg) for 5 days. Then, the Lin^−^ cells were enriched with the Lineage Cell Depletion Kit (130-090-858, Miltenyi Biotec) and infected with MSCVneo-MLL-AF9 retroviruses (*29*) and ipUSEPR-based dual sgRNAs (sgCtrl-dual and sgSgf29-dual; sequence shown in fig. S3B). Preleukemic cells were cultured in IMDM (Gibco) plus 15% FBS supplemented with murine SCF (20 ng/ml; PeproTech), murine IL-3 (10 ng/ml; PeproTech), and murine IL-6 (10 ng/ml; PeproTech) at 37°C. For the colony-forming assay, the transduced cells were seed at a density of 1 × 10^4^ per 35-mm culture dishes (27150, STEMCELL Technologies) in methylcellulose-based media (ColonyGEL 1201, ReachBio Research Labs) supplemented with murine recombinant IL-3 (10 ng/ml), IL-6, granulocyte-macrophage colony-stimulating factor, and murine recombinant SCF (50 ng/ml), along with G418 (1.0 mg/ml; Gibco) and/or puromycin (2 μg/ml). The colony cultures were incubated at 37°C for 6 to 7 days, and the blast-like colony-forming units were counted. For each passage, 1 × 10^4^ cells harvested from the colonies were subsequently replated in fresh methylcellulose media for up to three rounds. For in vivo leukemia engraftment assays, 0.8 million transduced preleukemic cells plus 1 million “helper” cells (BM mononuclear cells from B6.SJL CD45.1^+^ female mice) were resuspended in phosphate-buffered saline (PBS) and transplanted into lethally irradiated (900 cGy) 8- to 10-week-old CD45.1^+^ recipient mice via intravenous injection. Peripheral blood and spleen samples were collected from recipient mice at the same time point for each group (day 90 after transplantation). The cells were resuspended in ammonium chloride solution (07850, STEMCELL Technologies) to lyse the red cells, washed with PBS, and stained with FITC-CD45.2 (17-0454-82, eBioscience) for flow cytometry analysis. To examine the in vivo efficacy of Cpd_DC60, the mouse MLL-AF9 leukemic cells harvested from the secondary leukemia mice were transplanted into sublethally irradiated (320 cGy) 8- to 10-week-old CD45.1^+^ recipient mice via intravenous injection. Cpd_DC60 (custom synthesized by Ambinter Inc.) was first fully dissolved by dimethyl sulfoxide (DMSO) at 5 mg/ml and then diluted eightfolds by PBS for intraperitoneal injection. The leukemia recipient mice were given a vehicle or Cpd_DC60 (5 mg/kg body weight) treatment every other day from days 7 to 35 after transplantation. Peripheral blood, BM, and spleen samples were collected from recipient mice at the same time point for each group (day 36 after transplantation). All the mice were maintained on a 12-hour/12-hour light-dark cycle with food and water ad libitum. Mice were randomly assigned into each group. The recipient mice were euthanized by CO_2_ inhalation when signs of systemic illness appeared. All experiments on animals were performed in accordance with institutional guidelines and Institutional Animal Care and Use Committee (IACUC) protocol approved by City of Hope.

### “Human-in-mouse” leukemia model and in vivo bioluminescence imaging

The human-in-mouse xenograft leukemia model was established by transplanting 6- to 8-week-old NRGS mice [strain ID: NOD.Cg-*Rag1^tm1Mom^ Il2rg^tm1Wjl^* Tg(CMV-IL3,CSF2,KITLG)1Eav/J; RRID: IMSR_JAX:024099; the Jackson Laboratory] mice with human MOLM13-Cas9^+^/Luc^+^ cells. NRGS mice were randomly assigned into each group. MOLM13-Cas9^+^ human leukemic cells (AML) were transduced with pLenti CMV Puro LUC (17477, Addgene) lentiviruses and selected with puromycin (2 μg/ml) for 4 days to generate the luciferase-expressing cells (amenable to bioluminescence imaging). These MOLM13-Cas9^+^/Luc^+^ cells were then transduced with ipUSEPR-based dual sgRNAs (sgCtrl-dual and sgSGF29-dual; sequence shown in fig. S3B), pLVN vector (108101, Addgene), or pLVN-hSGF29_TST as indicated for each experimental group (Fig. 3A). To establish the leukemia model, 0.2 million transduced cells were resuspended in PBS and transplanted into 8- to 10-week-old NRGS recipient mice via intravenous injection. To monitor the leukemia progression, in vivo bioluminescence imaging was conducted on the recipient mice weekly. d-luciferin [4,5-dihydro-2-(6-hydroxy-2-benzothiazolyl)-4-thiazolecarboxylic acid potassium salt; LUCK-2G, GoldBio] was dissolved in PBS. Ten minutes before imaging, mice were weighed, injected with d-luciferin (150 mg/kg) via intraperitoneal injection and then anesthetized using isoflurane. Whole-body bioluminescence imaging was performed using a Lago X Imager (Spectral Instruments Imaging). The bioluminescence signal was presented in radiance in a unit of “photons/seconds/cm^2^/steradian.” The pseudocolor indicates the signal strength for leukemia burden. All the mice were maintained on a 12-hour/12-hour light-dark cycle with food and water ad libitum. The recipient mice were euthanized by CO_2_ inhalation when signs of systemic illness appeared. All experiments on animals were performed in accordance with institutional guidelines and IACUC protocol approved by City of Hope.

### Posttranslational modification mass spectrometry of histone

Five million sgCtrl or sgSgf29 transduced MLL-AF9-Cas9^+^ cells were harvested, washed once with PBS, and spun down at 500*g* for 5 min. The cell pellets were flash-frozen with dry ice and submitted for Mod Spec Service (Active Motif). Briefly, histones were acid-extracted, derivatized via propionylation, and digested with trypsin. The newly formed N termini were then propionylated, and the tryptic peptide samples were measured using a TSQ Quantum Ultra mass spectrometer coupled with an UltiMate 3000 Dionex nano-liquid chromatography system (Thermo Fisher Scientific). Each sample was measured with three technical replicates. The data were quantified using Skyline (*57*). The modification positions on histones that exhibit more than 0.015% of total histone in the sgCtrl sample were reported.

### SGF29-associated genomic DNA and H3K9ac ChIP-seq

Following procedures previously described in Li *et al.* (*21*), we incubated 10 million testing cells with 1% (v/v) formaldehyde at room temperature for 10 min, followed by the addition of 125 mM glycine to quench the excessive formaldehyde. The fixed cells were then washed twice with ice-cold PBS and resuspended in 250 μl of ChIP SDS lysis buffer [1% SDS, 10 mM EDTA, and 50 mM tris-HCl (pH 8.0)] supplemented with Halt Protease Inhibitor Cocktail (78430, Thermo Fisher Scientific). The lysed cells were sonicated by a Bioruptor (Diagenode) to shear the genomic DNA to ~150 to 300 bp in size and centrifuged at 10,000*g* for 5 min at room temperature, and the supernatant (containing the sheared chromatin) was mixed with the ChIP dilution buffer [0.01% SDS, 1.1% Triton X-100, 1.2 mM EDTA, 167 mM NaCl, and 16.7 mM tris-HCl (pH 8.0)] at 1:9 ratio. For detecting the SGF29-targeted genomic regions, the sheared chromatin sample from MLL-AF9-Cas9^+^ cells expressing pLVN-hSGF29_TST were captured by the MagStrep “type3” XT Beads (2-4090-002, IBA). For H3K9ac ChIP-seq, the sheared chromatin sample from MLL-AF9-Cas9^+^ cells expressing sgCtrl and sgSgf29 were incubated with the anti-H3K9ac antibody (1:100; 61663, Active Motif) at 4°C for overnight and captured by protein A/G magnetic beads (1:400; Dynabeads 10001D and 10003D, Invitrogen). The magnetic beads were washed with a low salt buffer [0.1% SDS, 1% Triton X-100, 2 mM EDTA, 150 mM NaCl, and 20 mM tris-HCl (pH 8.0)] followed by a high salt buffer [0.1% SDS, 1% Triton X-100, 2 mM EDTA, 500 mM NaCl, and 20 mM tris-HCl (pH 8.0)], a LiCl wash buffer [250 mM LiCl, 1% IGEPAL-CA630, 1% deoxycholic acid, 1 mM EDTA, and 10 mM tris-HCl (pH 8.0)], and the TE buffer [1 mM EDTA and 10 mM tris-HCl (pH 8.0)]. The washed beads were then incubated with reverse-crosslinking buffer (1.1% SDS, 110 mM sodium bicarbonate) at 65°C overnight, followed by GeneJET DNA purification (K0702, Thermo Fisher Scientific). The enriched genomic DNA was submitted for library prep and sequenced by a NovaSeq 6000 (paired-end 150 bp; ~20 million reads per sample) at Novogene Inc. The raw sequence reads were quality checked using the FASTQC software (version 0.11.8) and aligned against the mouse genome (mm10) using Burrows-Wheeler Aligner (version 0.7.17). The aligned reads were then sorted by Samtools (version 1.10), and the duplicated reads were removed by Picard MarkDuplicates (version 2.21.1). Peak-calling analysis to identify antibody-binding regions was performed using MACS2 (version 2.1.1), and the SPMR option was used to generate normalized pileup files for downstream analysis. ChIP-seq signals were calculated from the pileup files around TSS regions and visualized in plots using deepTools (version 3.5.1). Genes with more than 2.5-fold enrichment of TST-captured signal in the pLVN-hSGF29_TST sample over the input control sample at their TSS ± 1 kb regions were reported as high-confident SGF29 targets (*Rpl8* and *Rps2*). The profiles of SGF29-TST and H3K9ac ChIP-seq were visualized by IGV 2.14.0 (BROAD Institute).

### CRISPR-scan assisted drug discovery (CRISPR-SADD) workflow

#### Step 1—SGF29 CRISPR gene tiling scan and 2D CRISPR smoothening

The *Sgf29* CRISPR gene tiling scan library (147 sgRNAs targeting *Sgf29* coding exons) was delivered into the MLL-AF9-Cas9^+^ cells and processed as the methods shown for the Tudor domain–focused CRISPR screen. After the high-throughput sequencing, the NCS of individual sgRNA was processed by Gaussian kernel smoothing in R (*15*), and the average score over the trinucleotide codons was calculated for each peptide position.

#### Step 2—3D CRISPR mapping and ligand binding site prediction

Three-dimensional structural data of human SGF29-TTD in complex with H3(1-18)K4me3 (PDB ID: 3ME9) (*14*) was obtained from the Research Collaboratory for Structural Bioinformatics Protein Data Bank (RCSB PDB). Human SGF29-TTD structure was extracted from 3ME9 using PyMOL v2.0.4 (Schrödinger, LLC) and PDB2PQR server (*58*), and then the resultant pqr file was converted into the pdbqt format using AutoDockTools (*59*). Subsequently, the smoothened Sgf29 2D CRISPR-Scan scores (from the “Step 1” section) was mapped onto the SGF29-TTD 3D structures using the “Defined Attribute” and “Render by Attribute” functionalities in UCSF Chimera 1.15 (*60*). We then used PrankWeb (*33*) to computationally predict the druggable protein surface and overlapped with the CRISPR hypersensitive region of SGF29 to localize a 3D docking box suitable for the virtual compound screening.

#### Step 3—Virtual compound docking of the CRISPR-Scan–defined druggable pocket

Chemical structures were extracted from the ZINC15 database (http://zinc15.docking.org) (*35*), with the application of three selection filters of Protomers (i.e., chemical structures processed by the 3D molecule processing pipeline of ZINC15), Anodyne (i.e., protomers with no reactivity), and Ref (i.e., dominant chemical forms at pH 7.4). The 3D chemical structures were downloaded as mol2 from the following seven ZINC15 subsets: DrugBank Food and Drug Administration, DrugBank Investigational, Illuminating the Druggable Genome (IDG), National Cancer Institute (NCI) Plated 2007, Fluorochem, Maybridge, and ChemBridge. A total of ~1.6 million chemical mol2 files were split into subsets of 20,000 compounds in size using Open Babel v2.4.1 (*61*). Subsequently, each subset was converted into the pdbqt format (the input file format for AutoDock Vina) using PyRx v0.9.7 (*62*). Having both ligand and protein structures prepared for structure-based drug discovery, we used AutoDock Vina v1.1.2 (*34*), an in silico molecular docking program, to virtually dock these compounds into the docking box defined by Step 2 using City of Hope Saturn 2 Linux cluster. Last, the docking data were processed and exported to csv files using Raccoon2 (*59*), and 190 top candidate compounds from the docking results were selected for a cell-based survival screen.

### Molecular dynamics simulations

Top two conformations of Cpd_DC60 docked to SGF29-TTD using AutoDock Vina and the x-ray crystal structure of SGF29-TTD in complex with H3K4me3 peptide (PDB ID: 3ME9) were used for molecular dynamics simulations. Molecular dynamics simulations were performed using GROMACS (2019 package) with CHARMM36m force field, TIP3 water molecules, 0.15 mM sodium, and chloride ions were added to neutralize each system. The complexes were minimized in energy using the steepest descent method in GROMACS and equilibrated by performing 200 ps of molecular dynamics at 310 K using NVT (constant volume and constant temperature) ensemble. After that, a series of NPT (constant pressure and constant temperature) ensembles of 10 ns were performed with consecutive reduction of restraints from 5 to 1 kcal/mol per Å^2^ applied to all heavy atoms of protein. The final snapshot of the equilibration run was used as the starting structure of production simulations. We performed five replica runs with different initial velocities with each run up to 200 ns, providing a combined 1000-ns ensemble trajectory made of each velocity. To characterize the pairwise contacts made, we used the “get-contacts” python script library (www.github.com/getcontacts). The interaction energy between ligand-and-protein or ligand-and-aromatic ring residues (Tyr^238^, Tyr^245^, and Phe^264^) was calculated using the GROMACS “energy” module. The calculated interaction energy was used to identify which docked conformation of Cpd_DC60 on SGF29-TTD is the most energetically favorable over the endogenous ligand H3K4me3 peptide.

### Cell-based survival screen

One hundred ninety top candidate compounds suggested by CRISPR-SADD, together with 15 known compounds (serve as reference controls), were selected for functional validation. Compound information and source are listed in data S9. For Cpd_DC60, the initial small scale was obtained from the NCI Developmental Therapeutics Program (NCI/DTP) Open Chemicals Repository. Additional Cpd_DC60 was then custom synthesized by Ambinter (Orléans, France). MV4-11 cells were seeded at 20,000 cells per well, and U251 cells were seeded at 10,000 cells per well. Cells were cultured in 96-well plates at 100 μl per well and replated with fresh medium and compound every 3 days for up to 9 days. At each time point, 15 μl of the CellTiter-Glo 2.0 assay reagent (G9242, Promega) and 20 μl of resuspended cells were mixed in white flat-bottom 96-well plates (353296, Corning), and subsequently, the resultant luminescence was measured using an Infinite M1000 Pro plate reader (Tecan Trading AG, Switzerland). The relative CellTiter Glo signal was normalized to the control condition (DMSO) at each reading time point.

### AlphaScreen assay

AlphaScreen Histidine (Nickel Chelate) Detection Kit (6760619C, PerkinElmer) was used to determine the binding of SGF29-TTD and Cpd_DC60. The concentration of the purified SGF29-TTD was measured by NanoDrop One (Thermo Fisher Scientific) with an extinction coefficient of 31,400 M^−1^ cm^−1^. The H3(1-18)K4me3-bio (biotinylated H3K4me3) peptide was synthesized by Thermo Fisher Scientific. The AlphaScreen assay was set at a total reaction volume of 60 μl in white flat-bottom 96-well plates (353296, Corning), with the final concentrations of 75 nM SGF29-TTD, 150 nM H3(1-18)K4me3-bio, 0.8% DMSO, and acceptor/donor beads (20 μg/ml) in the reaction buffer [50 mM Hepes, 100 mM NaCl, and 5 mM TCEP (pH 7.4)]. Cpd_DC60 was first mixed with the H3(1-18)K4me3-bio peptide, then with the purified SGF29-TTD, follow by adding the acceptor/donor beads. The reaction mixture was incubated at 25°C in the dark with 100 rpm of agitation for 3 hours, and the AlphaScreen signals were measured using a BioTek Synergy Neo2 Hybrid Multimode Reader (Agilent Technologies) with an excitation wavelength of 680 nm and an emission detection wavelength of 615 nm.

### Code availability

The computational codes/tool packages used in this study include Genetic Perturbation Platform (BROAD Institute), Bowtie2 (Johns Hopkins University), UCSF Chimera 1.15 (UC San Francisco), Attune NxT v3.1.2 (Thermo Fisher Scientific), GSEA v4.1.0 (UC San Diego and BROAD Institute), FASTQC v0.11.8, Burrows-Wheeler Aligner v0.7.17, MACS2 v2.1.1, Samtools v1.10, STAR v2.6.1d, featureCounts v1.6.4, edgeR, deepTools v3.5.1, IGV 2.14.0 (BROAD Institute), Skyline, Picard MarkDuplicates v2.21.1, PyMOL v2.0.4 (Schrödinger, LLC), PDB2PQR server, AutoDockTools, PrankWeb, Open Babel v2.4.1, PyRx v0.9.7, AutoDock Vina v1.1.2, Raccoon2, GROMACS (2019 package), CHARMM36m force field, and Bio-Rad ChemiDoc MP (Bio-Rad). IC50 and two-sided Student’s *t* test were performed using Prism 9 (GraphPad).

### Human subjects/materials

*MLL*-r leukemia patient cells were obtained from pre-existing specimens from the previous publication (*41*). These biological specimens were not collected specifically for the current project through an interaction or intervention with living individuals. These specimens are not individually identifiable to the investigators, and the investigators have no access to link the samples to living individuals. The use of *MLL*-r leukemia patient cells was approved by the Institutional Review Board at City of Hope Cancer Center.
