# Supplementary material for: Therapeutic targeting Tudor domains in leukemia via CRISPR-Scan Assisted Drug Discovery
Source: Sci Adv. 2024 Feb 23;10(8):eadk3127. doi: 10.1126/sciadv.adk3127 (PMC10889360; doi:10.1126/sciadv.adk3127)
Supplement: Supplementary file 1 — Figs. S1 to S13 Table S1 Legends for data S1 to S10 [file sciadv.adk3127_sm.pdf]

Science Advances
AAAS

Supplementary Materials for

Therapeutic targeting Tudor domains in leukemia via CRISPR-Scan Assisted Drug Discovery

Anthony K.N. Chan *et al.*

Corresponding author: Chun-Wei Chen, cweichen@coh.org

Sci. Adv. **10**, eadk3127 (2024)
DOI: 10.1126/sciadv.adk3127

The PDF file includes:

Figs. S1 to S13
Table S1
Legends for data S1 to S10

Other Supplementary Material for this manuscript includes the following:

Data S1 to S10

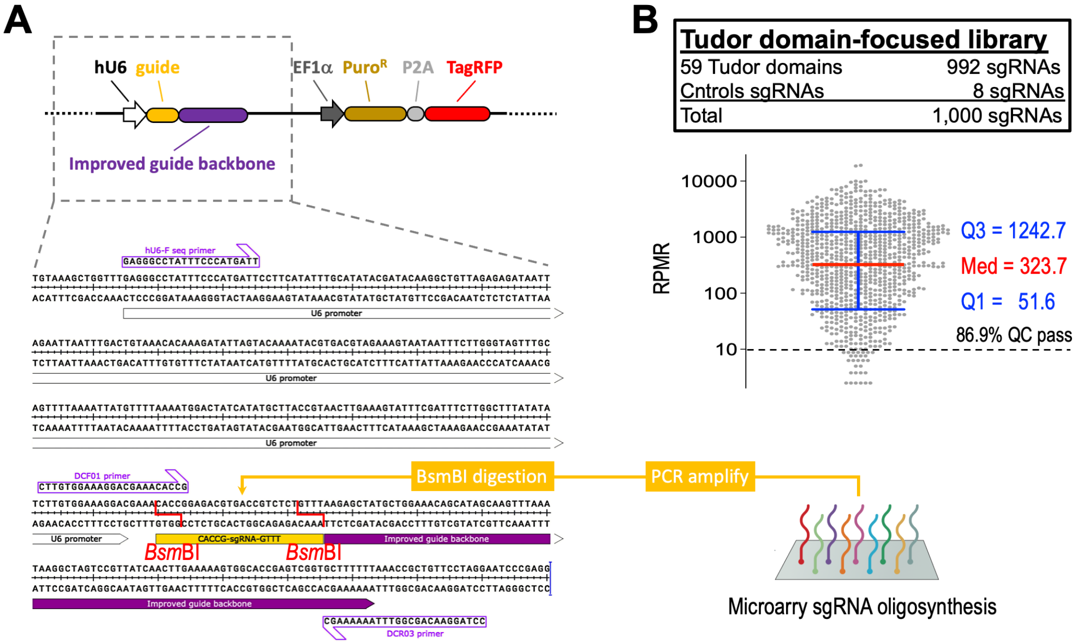

C

Gene	Tudor#	sgRNA#
Akap1	Tudor1	20
Alg13	Tudor1	13
Arid4a	Tudor1	13
Arid4b	Tudor1	10
Coil	Tudor1	26
Kdm4a	Tudor1	14
	Tudor2	19
Kdm4b	Tudor1	17
	Tudor2	23
Kdm4c	Tudor1	6
	Tudor2	12
Lbr	Tudor1	14
Lin9	Tudor1	15
Mtf2	Tudor1	12
Phf1	Tudor1	20
Phf19	Tudor1	18
Phf20	Tudor1	15
	Tudor2	7
Phf20l1	Tudor1	15
	Tudor2	9

Gene	Tudor#	sgRNA#
Rnf17	Tudor1	12
	Tudor2	10
	Tudor3	16
	Tudor4	10
Setdb1	Tudor1	11
	Tudor2	15
Sgf29	Tudor1	18
	Tudor2	41
Smn1	Tudor1	10
Smndc1	Tudor1	14
Snd1	Tudor1	20
Spin1	Tudor1	21
	Tudor2	17
	Tudor3	12
Stk31	Tudor1	7
Tdrd1	Tudor1	18
	Tudor2	12
	Tudor3	17
	Tudor4	13
Tdrd12	Tudor1	14

Gene	Tudor#	sgRNA#
Tdrd3	Tudor1	11
Tdrd5	Tudor1	18
Tdrd6	Tudor1	18
	Tudor2	18
	Tudor3	16
	Tudor4	14
	Tudor5	17
	Tudor6	19
	Tudor7	15
Tdrd7	Tudor1	10
	Tudor2	12
	Tudor3	14
Tdrd9	Tudor1	15
Tdrkh	Tudor1	19
Tnrc18	Tudor1	66
Trp53bp1	Tudor1	39
Uhrf1	Tudor1	36
Vwa3b	Tudor1	14
Zgpat	Tudor2	15

fig. S1. Tudor domain-focused CRISPR screen library used in this study.
(A) Map of the ipUSEPR vector expressing a sgRNA together with a puromycin-resistant gene (*Puro*^{R}) and a TagRFP fluorescent protein. Primers for Sanger (hU6-F_seq) and Illumina (DCF01 and DCR03) sequencing are listed. **(B)** Distribution of individual sgRNA frequencies RPMR (reads per million reads) in the Tudor domain-focused CRISPR library (total 1,000 sgRNAs). 86.9% of sgRNA in this library passed the QC by exhibiting frequency ≥ 10 RPMR. **(C)** Summary of the sgRNA distribution in the Tudor domain-focused CRISPR library targeting 59 Tudor domains (across 36 Tudor domain-containing genes) in the mouse genome.

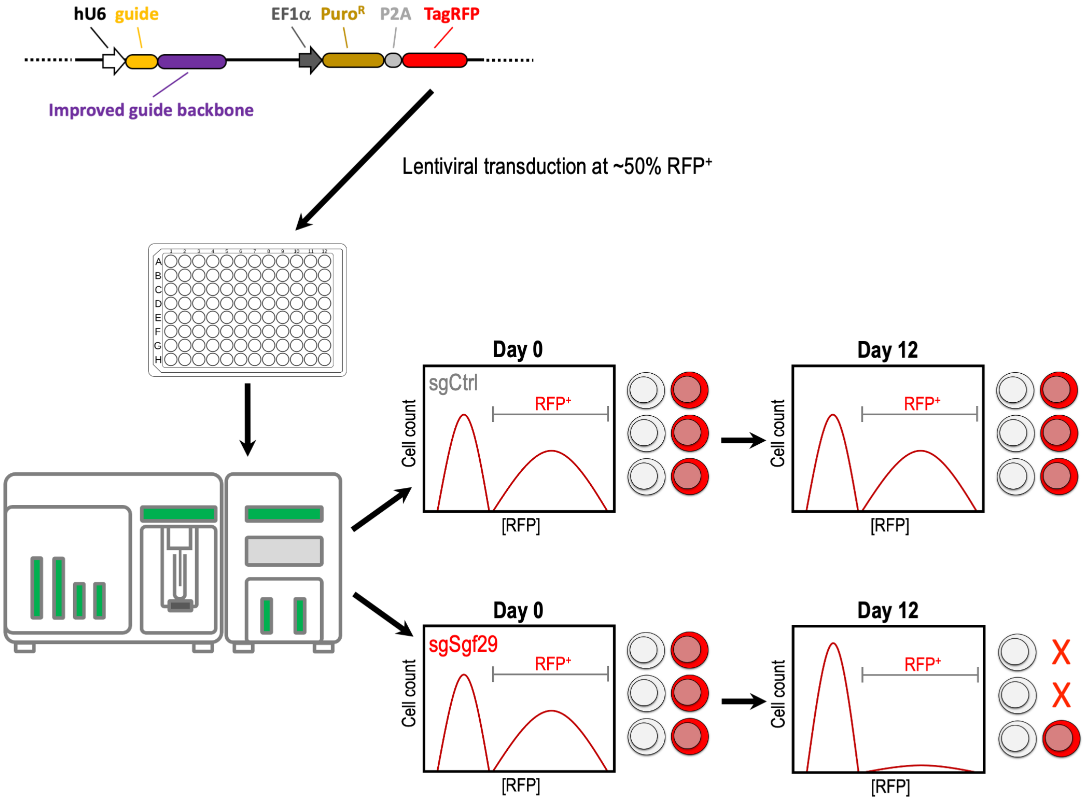

fig. S2. Schematic outline of the RFP flow cytometric growth competition assay.

A **Single sgRNA system:**

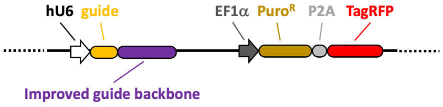

Single sgRNA used in this study:

sgRNA_ID	Guide sequence
sgCtrl#1	GATTCTAAAACGGATTACCA
sgCtrl#2	GGATGATAACTGGTCCGCAG
sgCtrl#3	GAAGATGGGCGGGAGTCTTC
sg_Sgf29#1	CCAGGTTTCCCGATCCAGAG
sg_Sgf29#2	TGTAACTGACTACTTCAGCT
sg_Sgf29#3	CATAAGAGGTGTCTTCAAAC
sg_MYC	GCCGTATTTCTACTGCGACG
sg_SGF29#1	TGTGGCTCAGAGATACGTGG
sg_SGF29#2	CGGTAGAAGCAGGTAGTCTG
sg_SGF29#3	AGCAGGTAGTCTGGGGATAC
sg_SGF29#4	GATCAGGGCGCGGTAGAAGC
sgKat2a#1	CCACCTGATGAACCACCTAA
sgKat2a#2	TATGCTGACGAGTACGCCAT
sgKat2b#1	CATACTCATCTGCATATGTG
sgKat2b#2	TATGCAGATGAGTATGCCAT
sg_mRpl8#1	GCGCACGTGAAGCACCGTAA
sg_mRpl8#2	GACTGTGGCGTAGTTCCCGG
sg_mRps2#1	AGGTAAAGCTGAAGACAAGG
sg_mRps2#2	ATACCTACCAAAGTTGCCCA

B **Dual sgRNA system:**

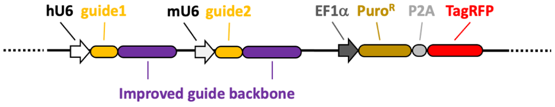

Dual sgRNA used in Figure 2:

sgRNA_ID	Guide 1 sequence	Guide 2 sequence
sgCtrl-dual	GATTCTAAAACGGATTACCA	GGATGATAACTGGTCCGCAG
sgSgf29-dual	TGTAACTGACTACTTCAGCT	CATAAGAGGTGTCTTCAAAC

Dual sgRNA used in Figure 3:

sgRNA_ID	Guide 1 sequence	Guide 2 sequence
sgCtrl-dual	GAAGATGGGCGGGAGTCTTC	GGATGATAACTGGTCCGCAG
sgSGF29-dual	CGGTAGAAGCAGGTAGTCTG	TGTGGCTCAGAGATACGTGG

fig. S3. Single and dual sgRNA sequences used in this study.

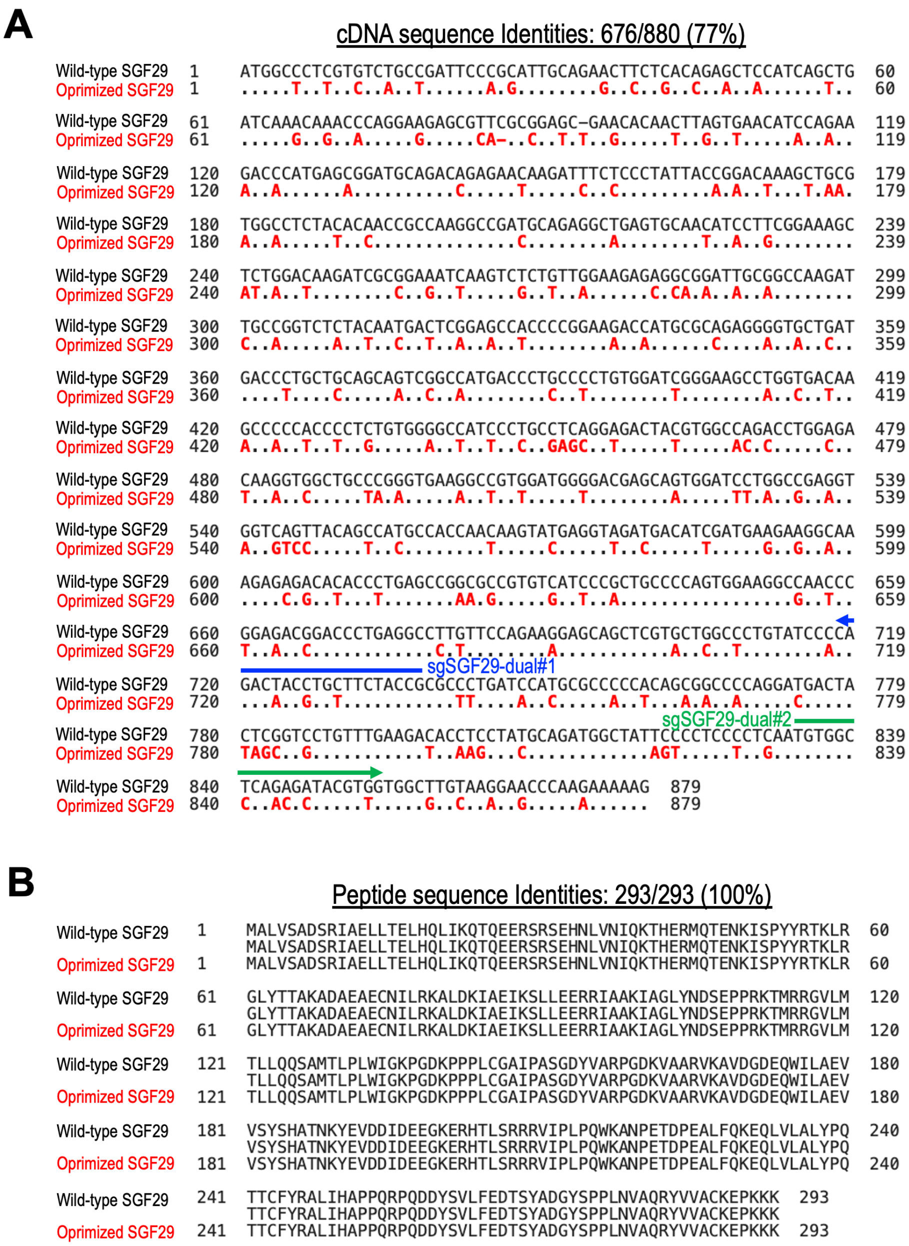

fig. S4. Design of the optimized SGF29 cDNA construct.

(A) The cDNA sequence of the optimized SGF29 construct (contains synonymous mutations) shares 77% identity with the wild-type human *SGF29* cDNA. The switched nucleotides in the optimized *SGF29* cDNA are labeled (red), which facilitates the protein expression via optimizing the codon usage (CLC Genomics Workbench, QIAGEN). The optimized SGF29 cDNA also introduces miss-matched sequences within the sgSGF29-dual #1 (blue) and #2 (green) landing sites, thus, allowing reconstitution of SGF29 protein expression in the sgSGF29-dual targeted cells shown in Fig. 3. **(B)** The peptide sequence of the optimized SGF29 construct shares 100% identity with the wild-type human SGF29 peptide.

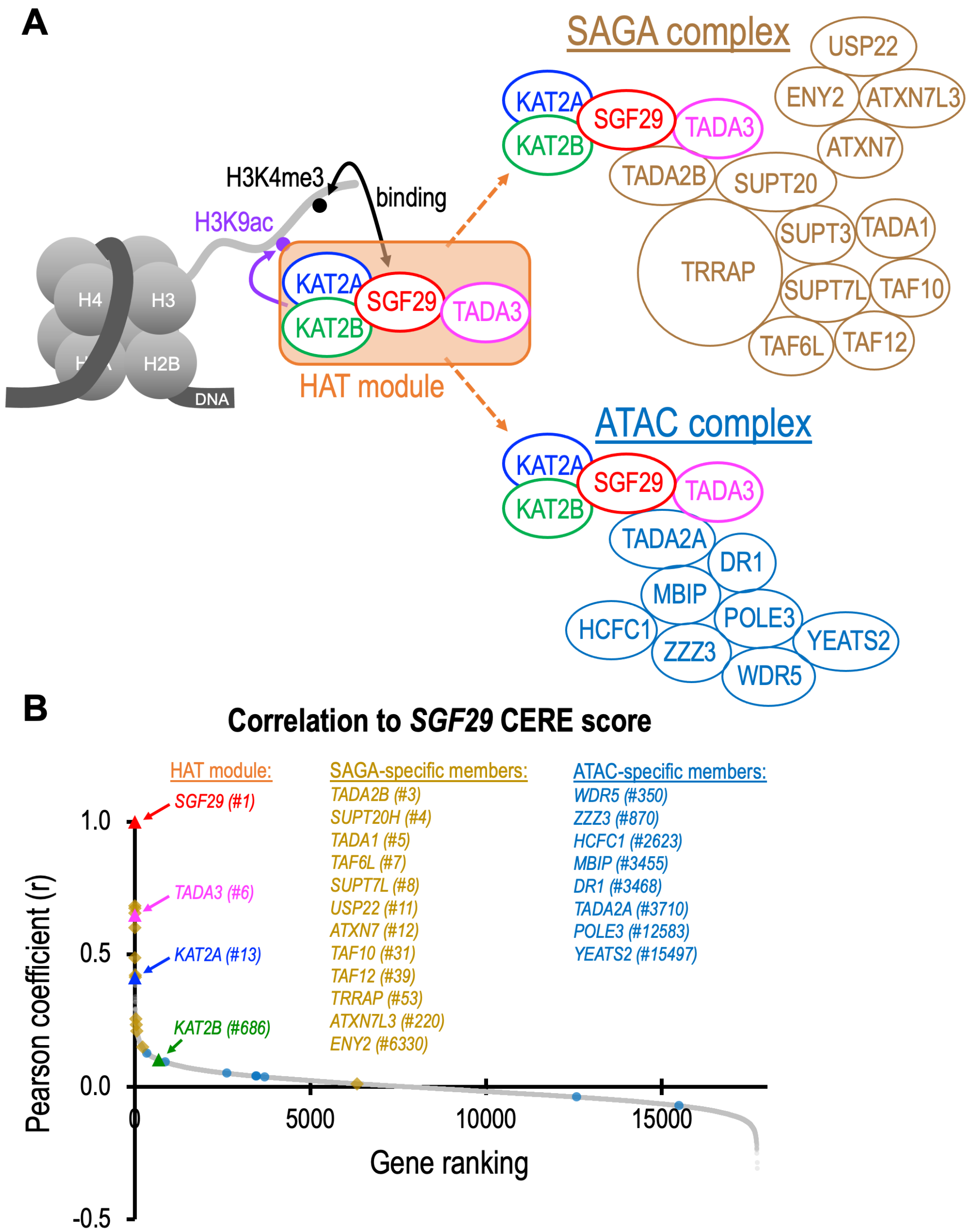

fig. S5. Participation of SGF29 in SAGA and ATAC complexes.
(A) Schematic outline of SAGA and ATAC complexes. **(B)** Gene ranking based on the Pearson coefficient (r) of the CERE scores between *SGF29* and a total of 17,709 genes examined in the DepMap genome-wide CRISPR screen consortium database (source: https://depmap.org/portal/; BROAD Institute). The SAGA-specific (dark yellow) and ATAC-specific (dark blue) members are labeled. In addition, there are four overlapped members, including SGF29 (red), TDAD3 (pink), KAT2A (blue), and KAT2B (green), formed the histone acetyltransferase (HAT) module (orange box in panel A) that participate in both SAGA and ATAC complexes.

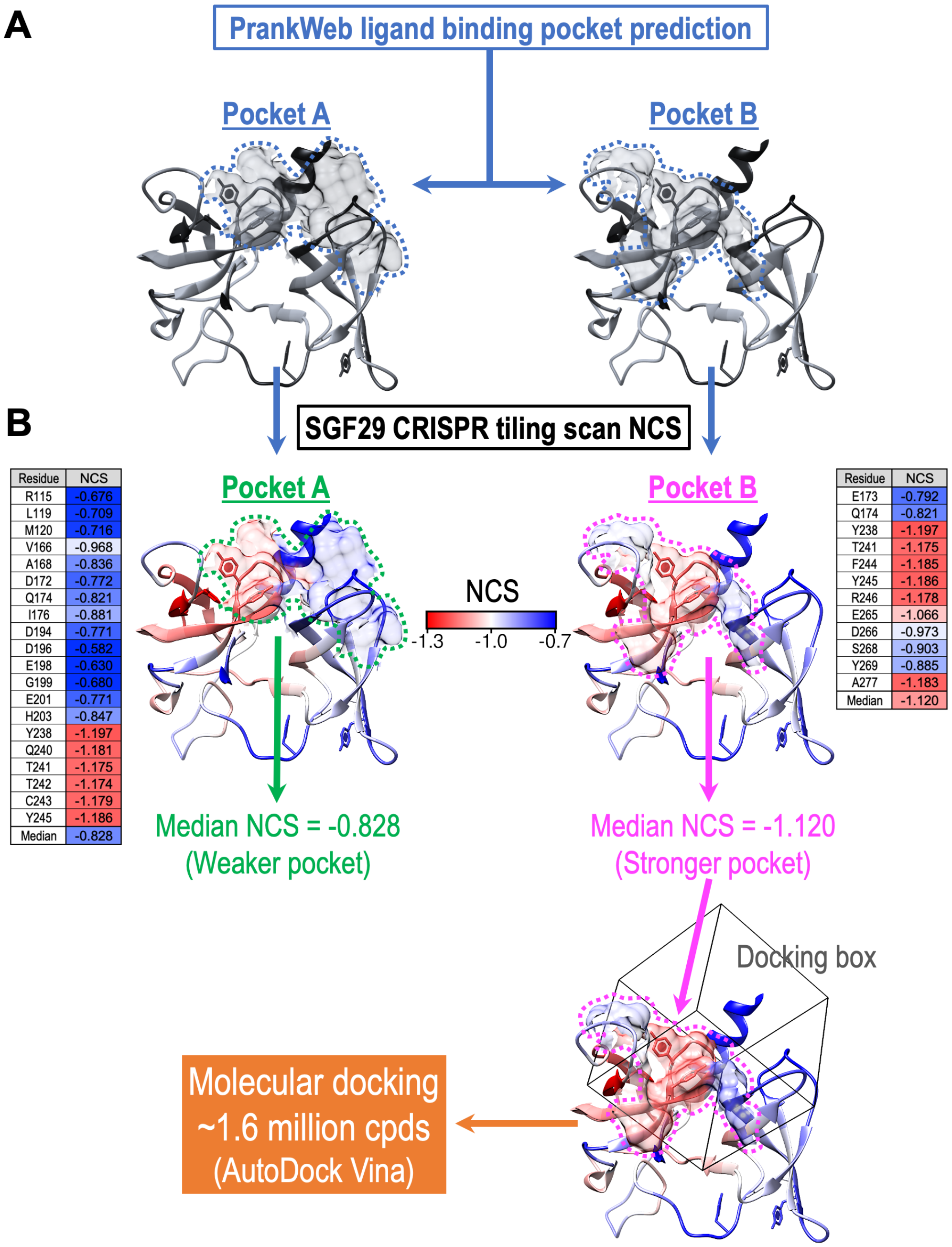

fig. S6. Determination of the SGF29 surface pocket for molecular docking.
(A) PrankWeb server predicted top two ligandable protein surface areas, Pocket A and Pocket B, on the SGF29 TTD domain (PDB ID: 3ME9). **(B)** Evaluation of the SGF29 CRISPR tiling scan revealed a stronger median NCS score in Pocket B (-1.120) than Pocket A (-0.828), indicating Pocket B as a more essential surface area on SGF29-TTD for therapeutic targeting.

A

#	MV4-11 d3	MV4-11 d6	MV4-11 d9	U251 d3	U251 d6	U251 d9	Cpd_Name	Δ Score (%)
	0.4	0.2	0.2	14.5	0.8	0.2	I-BET151	(0.0)
	0.3	0.2	0.3	17.0	0.6	0.1	JQ1	(0.1)
#1	69.0	5.0	0.3	95.0	105.6	102.3	EPZ-5676	(-102.1)
#2	2.4	0.2	0.1	96.7	102.5	96.8	**Cpd_DC60**	**(-96.7)**
#3	30.0	15.4	2.5	89.2	100.3	99.1	**Cpd_DC201**	**(-96.6)**
#4	46.0	14.5	2.8	90.7	94.0	81.9	**Cpd_DC11**	**(-79.1)**
#5	33.7	6.3	0.0	83.0	89.7	73.6	**Cpd_DC157**	**(-73.6)**
#6	24.0	1.9	0.5	91.3	68.3	66.2	**Cpd_DC182**	**(-65.7)**
#7	20.3	1.3	0.2	83.5	71.4	55.1	**Cpd_DC21**	**(-54.9)**
#8	41.1		1.6	93.7	76.2	55.1	Cpd_DC16	(-53.5)
#9	25.3	7.6	9.5	67.3	49.5	46.8	Cpd_DC176	(-37.2)
#10	25.4	10.3	5.0	55.9	55.7	37.4	Cpd_DC165	(-32.4)
#11	23.1	7.1	2.1	71.4	51.0	30.7	Cpd_DC171	(-28.7)
#12	68.8	36.0	5.0	82.5	46.1	26.2	OICR-9429	(-21.3)
#13	29.1	11.0	2.8	49.6	42.8	23.3	Cpd_DC160	(-20.5)
#14	17.9	0.4	0.2	57.0	28.8	15.8	A-485	(-15.7)
#15	51.5	3.4	0.2	59.8	29.4	15.7	Cpd_DC244	(-15.5)
#16	0.1	0.0	0.0	61.2	33.2	14.6	Cpd_DC196	(-14.6)
#17	89.6	39.5	6.0	92.2	59.4	19.8	A-366	(-13.8)
#18	33.3	11.8	1.4	50.7	28.5	14.7	Cpd_DC148	(-13.3)
#19	41.6	14.8	4.4	87.4	48.7	11.0	Cpd_DC23	(-6.5)

BET inhibitor *Dawson et al. (36)* (I-BET151)
BRD4 inhibitor *Zuber et al. (10)* (JQ1)
DOT1L inhibitor *Daigle et al. (37)* (EPZ-5676)
Candidate compounds (#2–#7)
WDR5 inhibitor *Grebien et al. (38)* (OICR-9429)
P300/CBP inhibitor *Lasko et al. (39)* (A-485)
G9a inhibitor *Pappano et al. (40)* (A-366)

B

Cpd Name	ZINC ID	ΔG° (kJ/mol)*
Cpd_DC60	ZINC000090751663	-9.7
Cpd_DC201	ZINC000102713262	-9.5
Cpd_DC11	ZINC000065362194	-10.3
Cpd_DC157	ZINC000102597834	-9.7
Cpd_DC182	ZINC000029064337	-9.6
Cpd_DC21	ZINC000012244660	-10.2

*Predicted by AutoDock Vina

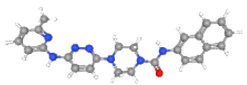

Cpd_DC11 ($C_{25}H_{25}N_7O$)
Mwt. 439.5 g/mol
ZINC000065362194

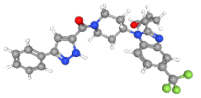

Cpd_DC21 ($C_{27}H_{26}F_3N_5O_2$)
Mwt. 509.5 g/mol
ZINC000012244660

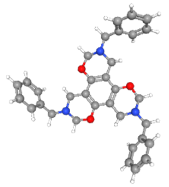

Cpd_DC60 ($C_{33}H_{33}N_3O_3$)
Mwt. 519.6 g/mol
ZINC000090751663

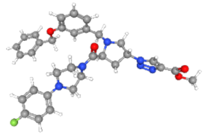

Cpd_DC157 ($C_{33}H_{35}FN_6O_4$)
Mwt. 598.7 g/mol
ZINC000102597834

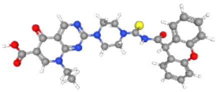

Cpd_DC182 ($C_{29}H_{26}N_6O_5S$)
Mwt. 570.6 g/mol
ZINC000029064337

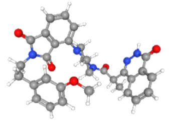

Cpd_DC201 ($C_{32}H_{31}N_5O_5$)
Mwt. 565.6 g/mol
ZINC000102713262

fig. S7. Information of the candidate SGF29 targeting compounds.
(A) Detail CellTiter Glo data of the MV4-11 selective group (19 cpds) from Fig. 5E. Four reference compounds from the MV4-11 selective group (EPZ-5676, OICR-9429, A-485, and A-366) and two from the general toxic group (I-BET151 and JQ1) are labeled in green. Δ Score indicates CellTiter Glo % ([MV4-11] – [U251]) on day 9. Six compounds with stronger MV4-11 selective killing effects (i.e., lower Δ Score) are selected as candidate SGF29 inhibitors (highlighted in black). **(B)** ZINC database ID, predicted binding free energy (ΔG°) to SGF29-TTD, and the chemical structure of each candidate SGF29 inhibitor are indicated with H (white), C (grey), N (blue), O (red), F (green), and S (yellow).

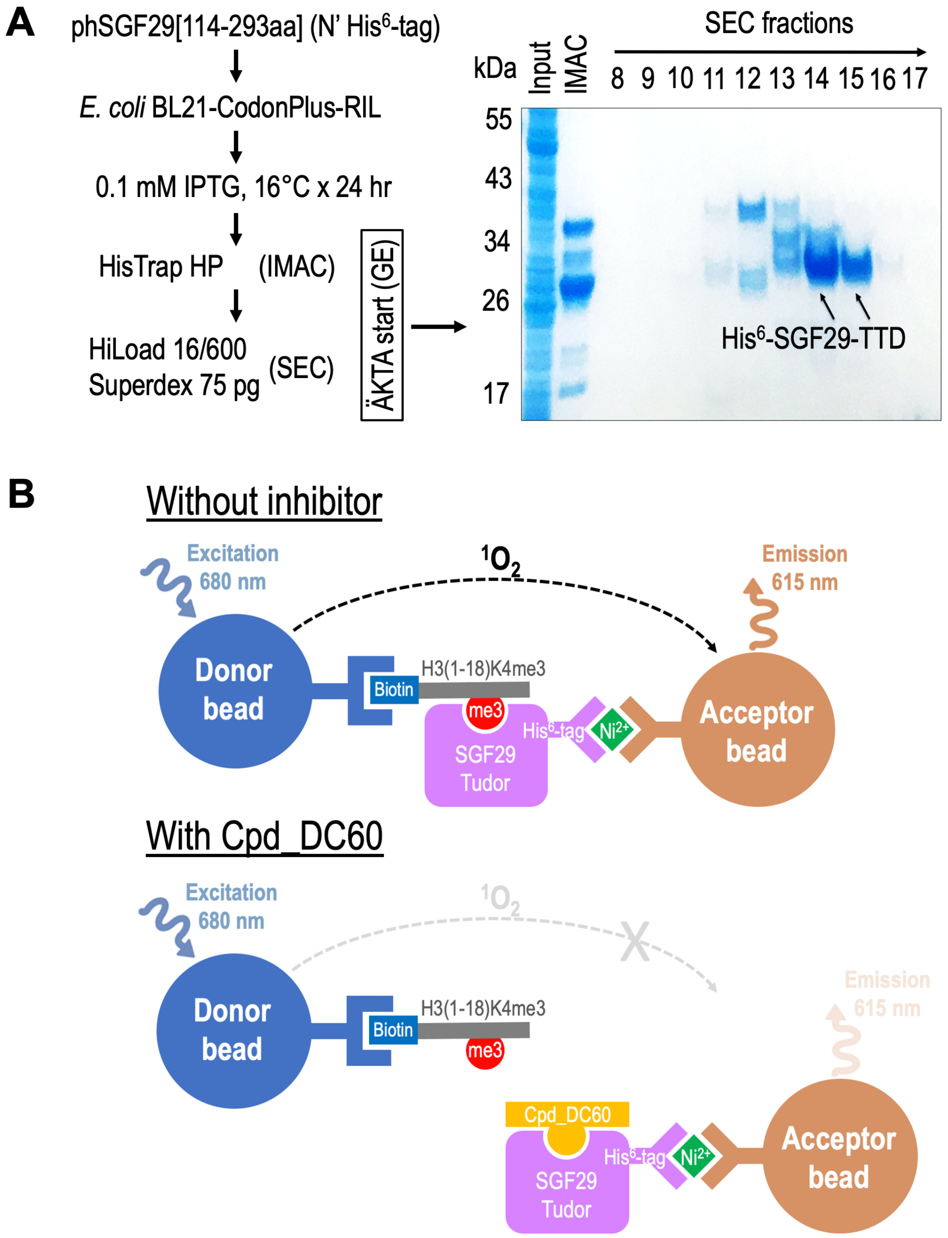

fig. S8. AlphaScreen assay for evaluating the SGF29 Tudor domain inhibitor.
(A) Purification of bacterial expressed recombinant human SGF29-TTD peptide (N' His^6-tagged) using immobilized metal affinity chromatography (IMAC) and size exclusion chromatography (SEC). **(B)** Schematic outline of AlphaScreen assay based on the interaction of SGF29-TTD with its natural ligand H3K4me3 peptide.

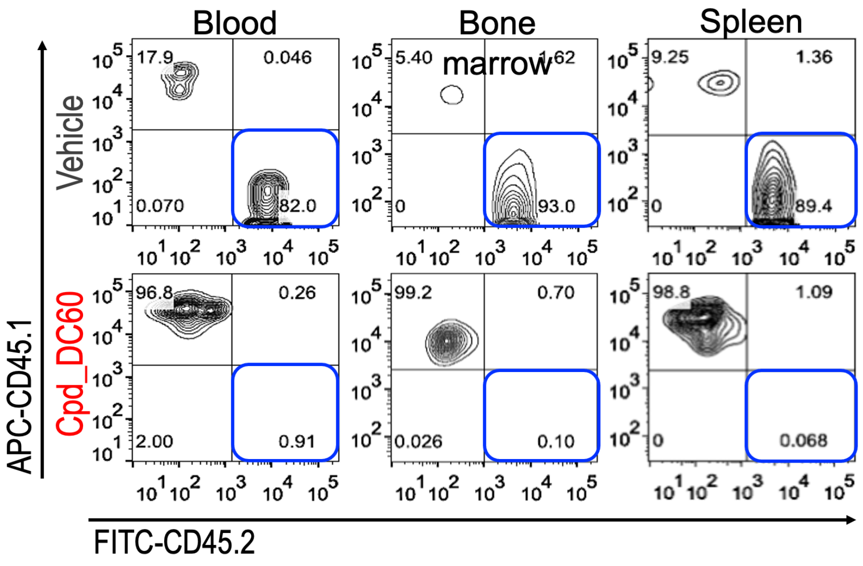

fig. S9. Analysis of CD45.2^{+} leukemia engraftment using flow cytometry.
Representative CD45.1 and CD45.2 flow cytometric profiles in the peripheral blood, bone marrow, and spleen of the CD45.1^{+} recipient mice with or without Cpd_DC60 treatment.

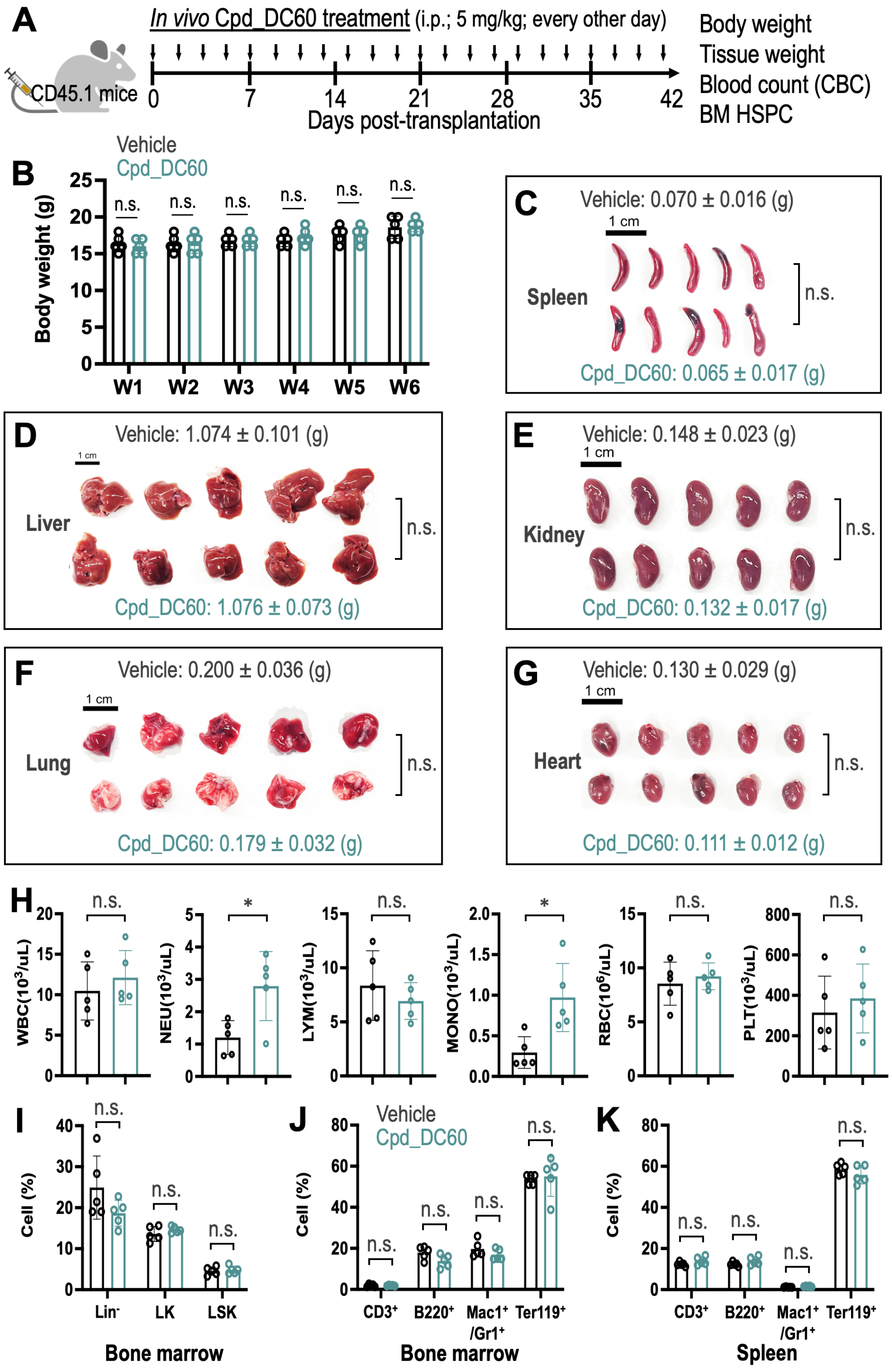

fig. S10. Toxicity evaluation of *in vivo* Cpd_DC60 treatment.
(A) Schematic outline of a 6-week long *in vivo* toxicity evaluation with alternate-day dosing of 5 mg/kg Cpd_DC60 in C57BL/6 CD45.1 mice (n = 5 mice per group). **(B)** Mouse body weight, **(C-G)** organ morphology and size, and **(H)** complete blood count of peripheral blood cell populations after six weeks of Cpd_DC60 treatment. **(I)** Lineage-negative (Lin^-), hematopoietic progenitor (LK), and hematopoietic stem/progenitor (LSK) cells in bone marrow, and **(J, K)** T cells ($CD3^+$), B cells ($B220^+$), myeloid cells ($Mac1^+/Gr1^+$), and red blood cells ($Ter119^+$) in bone marrow and spleen. Data are represented as mean ± SD. *$P < 0.05$ by two-sided Student's t-test.

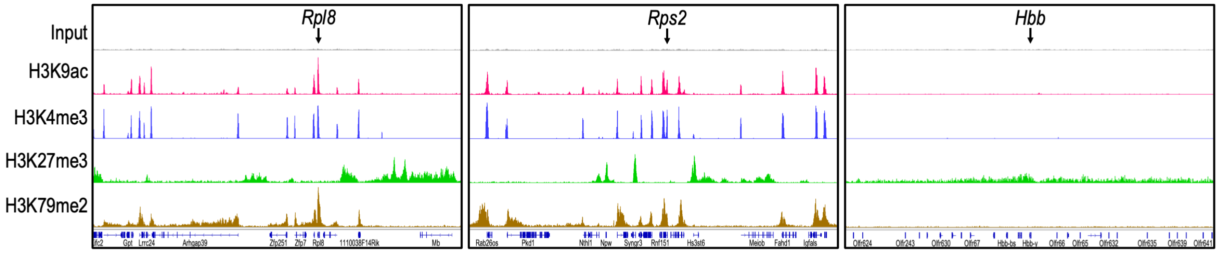

fig. S11. Chromatin distribution of H3K9ac and H3K4me3.
ChIP-seq profiles of H3K9ac (red), H3K4me3 (blue) at (left) *Rpl8*, (mid) *Rps2*, and (right) *Hbb* loci in mouse MLL-AF9 cells. The suppressed vs. expressed euchromatin regions are indicated by the silent (H3K27me3, green) and active (H3K79me2, brown) histone marks.

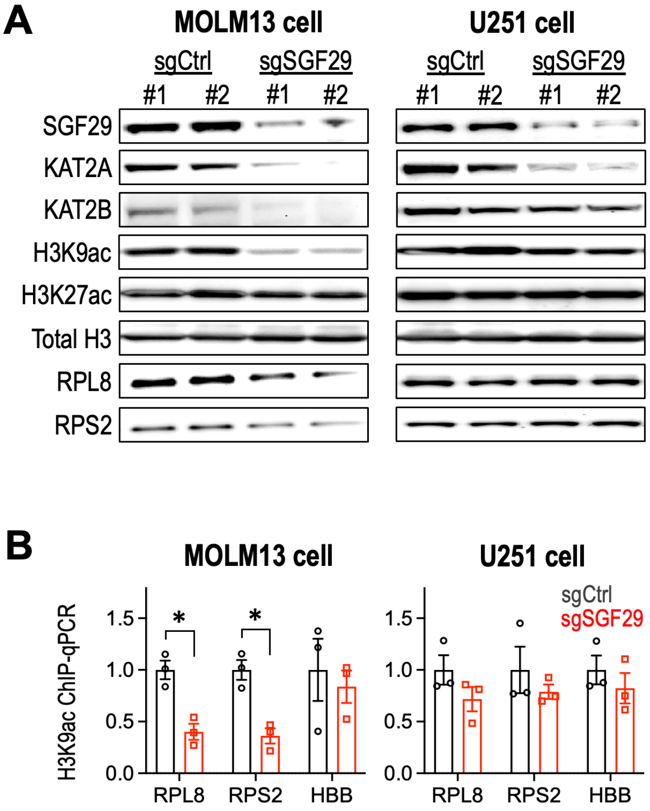

fig. S12. Differential regulation of SGF29 pathway in MOLM13 and U251 cells.
(A) Western blot of SGF29, KAT2A, KAT2B, H3K9ac, H3K27ac, total H3, RPL8, RPS2 and (B) H3K9ac ChIP-qPCR in SGF29-dependent (MOLM13 leukemia) and SGF29-independent (U251 glioblastoma) human cell lines.

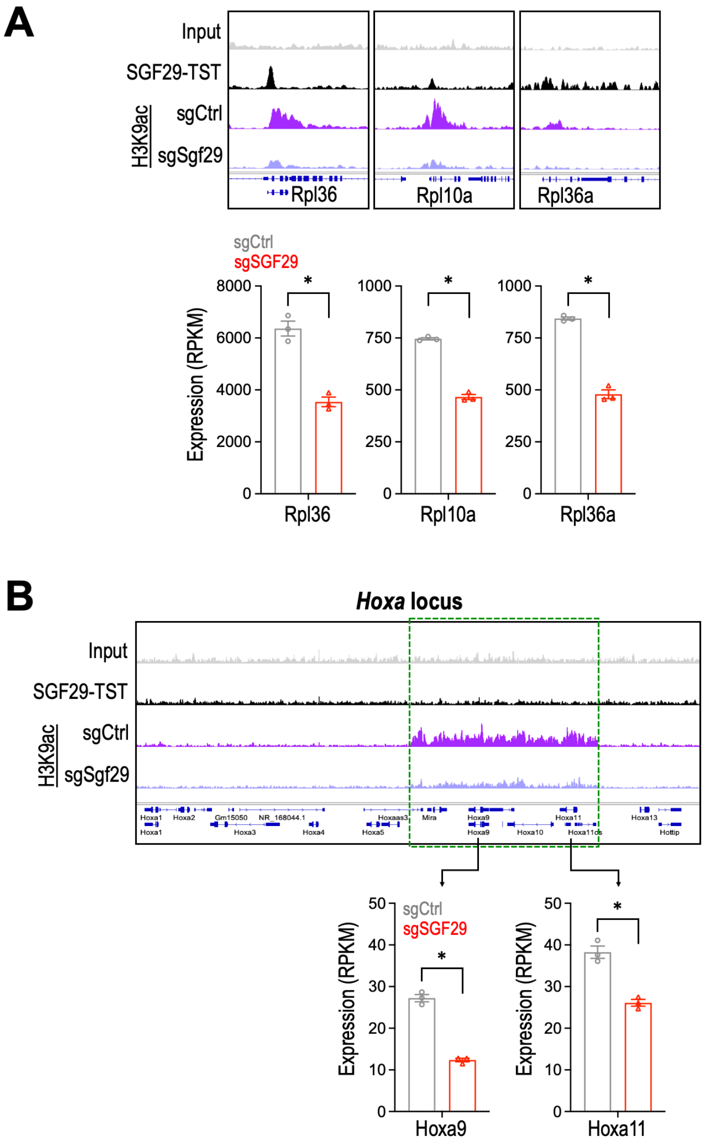

fig. S13. Effect of SGF29 on additional chromatin loci.
Distribution of SGF29-TST and H3K9ac ChIP-seq signal at **(A)** *Rpl36*, *Rpl10a*, *Rpl36a* and **(B)** *Hoxa* loci in mouse MLL-AF9 leukemia cells.

table S1. *MLL*-r leukemia patient cells used in this study.

Patient ID	TYPE	VARIANT	GENE	PROTEIN CHANGE	CDS CHANGE	COVERAGE	% READS	CNA-TYPE	RE-GENE2	RE-DESCRIPTION
042	somatic	RE	KMT2A						AFF1	fusion
	somatic	RE	MLL						MAPK10	RE
	VUS	SNV	FAM123B	Y599C	1796A>G	432	100%			
	VUS	SNV	MTOR	G307R	919G>A	577	2%			
	somatic	SNV	NRAS	G13D	38G>A	505	33%			
	VUS	SNV	RAF1	S301I	902G>T	674	16%			
	VUS	SNV	RARA	P440L	1319C>T	377	49%			
	VUS	SNV	SMARCA4	R359Q	1076G>A	396	53%			
	VUS	SNV	SPEN	I2469V	7405A>G	830	46%			
	VUS	SNV	TGFBR2	V387M	1159G>A	674	49%			
	somatic	SNV	TP53	G154S	460G>A	662	50%			
	somatic	SNV	TP53	N239D	715A>G	500	0%			
	somatic	SNV	TP53	R175H	524G>A	682	2%			
	somatic	SNV	TP53	R248W	742C>T	663	1%			
	somatic	SNV	TP53	T256A	766A>G	500	0%			
	VUS	SNV	TSHR	I155L	463A>T	399	47%			
049	somatic	InDel	CREBBP	Q1079fs*8	3234_3235insT	418	23%			
	somatic	InDel	ETV6	F102fs*21	305_306insGT	357	24%			
	VUS	InDel	PCLO	S4296_S4297insSRPS	12888_12889insTCCAGACCTTCC	653	26%			
	somatic	RE	MLL						AFF1	fusion
	somatic	SNV	CHEK1	W328L	983G>T	518	0%			
	somatic	SNV	CREBBP	S381*	1142C>A	500	0%			
	somatic	SNV	CREBBP	splice	3836+1G>A	424	11%			
	VUS	SNV	CREBBP	R714H	2141G>A	456	44%			
	VUS	SNV	CSF1R	E557K	1669G>A	501	47%			
	VUS	SNV	EPHA5	H329N	985C>A	501	0%			
	VUS	SNV	EPHB1	R367H	1100G>A	514	43%			
	VUS	SNV	IRS2	H1147Q	3441C>G	421	51%			
	somatic	SNV	MAP3K13	splice	6601G>T	500	0%			
	VUS	SNV	PARP3	E476K	1426G>A	288	51%			
	VUS	SNV	PARP4	V459A	1376T>C	249	49%			
	VUS	SNV	PRDM1	R302P	905G>C	591	52%			
	VUS	SNV	RAD51L3	L53M	157C>A	295	0%			
	VUS	SNV	ROS1	V849F	2545G>T	440	52%			
	VUS	SNV	SMARCA1	P840T	2518C>A	699	35%			
072	somatic	CNA	CDKN2A					loss		
	somatic	CNA	CDKN2B					loss		
	somatic	RE	KMT2A						AFF1	fusion
	VUS	SNV	DDR2	R668H	2003G>A	685	55%			
	VUS	SNV	FANCM	V1336D	4007T>A	710	48%			
	VUS	SNV	GATA2	P161A	481C>G	631	49%			
	VUS	SNV	JAK3	R432C	1294C>T	497	50%			
	VUS	SNV	KDM6A	T584M	1751C>T	814	50%			
	somatic	SNV	KRAS	G12S	34G>A	723	2%			
	VUS	SNV	MAGED1	A101T	301G>A	581	52%			
	VUS	SNV	MLL3	L3311F	9931C>T	804	49%			
	VUS	SNV	NF1	R2269H	6806G>A	511	48%			
	somatic	SNV	NRAS	G12S	34G>A	650	17%			
	somatic	SNV	NRAS	Q61R	182A>G	668	1%			
	VUS	SNV	PARP3	A33G	98C>G	614	48%			
	somatic	SNV	PIK3CA	E545K	1633G>A	498	3%			
	VUS	SNV	TCF3	R371*	1111C>T	726	12%			
	VUS	SNV	TCF3	P4L	11C>T	681	50%			

Leukemia patient cells reported in *Wang et al. (41)*

Supplementary excel tables:

Data S1 **List of Tudor domains and Tudor domain-containing genes.**
Data S2 **QC sequencing of the Tudor domain-focused CRISPR library.**
Data S3 **High-throughput sequencing of the Tudor domain-focused CRISPR screen (RPMR).**
Data S4 **Histone modification mass spec data table.**
Data S5 **List of Sgf29-regulated genes.**
Data S6 **SGF29 binding signal at the TSS +/- 1kb of the Sgf29-regulated genes.**
Data S7 **QC sequencing of the Sgf29 CRISPR tiling scan library.**
Data S8 **High-throughput sequencing of the Sgf29 CRISPR tiling scan (RPMR).**
Data S9 **Information of the compounds selected for CellTiter Glo assay in MV4-11 and U251 cells.**
Data S10 **Normalized CRISPR score (NCS) of Dot1l, Mof, and Lsd1 CRISPR tiling in mouse MLL-AF9 leukemia cells.**
